# Investigating Mitonuclear Genetic Interactions Through Machine Learning: A Case Study on Cold Adaptation Genes in Human Populations From Different European Climate Regions

**DOI:** 10.3389/fphys.2020.575968

**Published:** 2020-11-11

**Authors:** Alena Kalyakulina, Vincenzo Iannuzzi, Marco Sazzini, Paolo Garagnani, Sarika Jalan, Claudio Franceschi, Mikhail Ivanchenko, Cristina Giuliani

**Affiliations:** ^1^Department of Applied Mathematics, Institute of Information Technologies, Mathematics and Mechanics, Lobachevsky State University of Nizhny Novgorod, Nizhny Novgorod, Russia; ^2^Alma Mater Research Institute on Global Challenges and Climate Change (Alma Climate), University of Bologna, Bologna, Italy; ^3^Laboratory of Molecular Anthropology and Centre for Genome Biology, Department of Biological, Geological and Environmental Sciences, University of Bologna, Bologna, Italy; ^4^Department of Experimental, Diagnostic and Specialty Medicine (DIMES), University of Bologna, Bologna, Italy; ^5^Complex Systems Laboratory, Discipline of Physics, Indian Institute of Technology Indore, Indore, India; ^6^Center for Theoretical Physics of Complex Systems, Institute for Basic Science (IBS), Daejeon, South Korea; ^7^Laboratory of Systems Medicine of Healthy Aging, Lobachevsky State University of Nizhny Novgorod, Nizhny Novgorod, Russia; ^8^School of Anthropology and Museum Ethnography, University of Oxford, Oxford, United Kingdom

**Keywords:** mitonuclear interactions, human populations, cold adaptation, machine learning, human ecology, human evolution

## Abstract

Cold climates represent one of the major environmental challenges that anatomically modern humans faced during their dispersal out of Africa. The related adaptive traits have been achieved by modulation of thermogenesis and thermoregulation processes where nuclear (nuc) and mitochondrial (mt) genes play a major role. In human populations, mitonuclear genetic interactions are the result of both the peculiar genetic history of each human group and the different environments they have long occupied. This study aims to investigate mitonuclear genetic interactions by considering all the mitochondrial genes and 28 nuclear genes involved in brown adipose tissue metabolism, which have been previously hypothesized to be crucial for cold adaptation. For this purpose, we focused on three human populations (i.e., Finnish, British, and Central Italian people) of European ancestry from different biogeographical and climatic areas, and we used a machine learning approach to identify relevant nucDNA–mtDNA interactions that characterized each population. The obtained results are twofold: (i) at the methodological level, we demonstrated that a machine learning approach is able to detect patterns of genetic structure among human groups from different latitudes both at single genes and by considering combinations of mtDNA and nucDNA loci; (ii) at the biological level, the analysis identified population-specific nuclear genes and variants that likely play a relevant biological role in association with a mitochondrial gene (such as the “obesity gene” *FTO* in Finnish people). Further studies are needed to fully elucidate the evolutionary dynamics (e.g., migration, admixture, and/or local adaptation) that shaped these nucDNA–mtDNA interactions and their functional role.

## Introduction

The mitonuclear interaction can be considered the most successful mutualism in the history of life according to the complex dynamics of conflicts and cooperation that has been established between the mitochondrial and nuclear genomic backgrounds (Rand and Mossman, 2020). In fact, mitochondria and nucleus communicate to ensure optimal cellular function, and a part of the mitochondrial proteins are encoded in the nucleus. This communication occurs at many levels (Mottis et al., 2019).

Mitochondria actively influence and physically interact with other cellular components, such as the lysosomes, the endoplasmic reticulum, and cytosolic pathways, creating a mitocellular communication network based on a variety of signals. Then, small molecules (e.g., AMP, NAD^+^, ROS, oxygen, and other metabolites) act as mitochondrial messengers, and they signal mitochondrial activity to other cellular components. Mitochondria communicate also with distant tissues through circulating molecules, called mitokines (e.g., FGF21 and GDF15), which are nuclear-encoded signaling molecules secreted by cells that experienced mitochondrial stress.

Another form of communication – which is the one here analyzed – occurs at the genomic level (Mottis et al., 2019). It is estimated that the coevolution between mitochondrial and nuclear genomes has occurred for more than 1.5 billion years (Martijn et al., 2018), and it is likely that different mitonuclear interactions may contribute to enable organisms’ adaptation to changing environments (“mitonuclear ecology”) (Hill, 2015). Natural selection may favor tandem changes, creating coadapted mitonuclear genotypes (Rand et al., 2004; Burton et al., 2013; Wolff et al., 2014; Hill, 2015). Moreover, much evidence of coevolution between mitochondria and nuclear genomes have been described in natural populations, suggesting that tandem changes appear to be consistent with a model of compensatory mitonuclear coevolution (Osada and Akashi, 2012; van der Sluis et al., 2015; Sloan et al., 2017; Barreto et al., 2018; Hill et al., 2019).

Recent studies demonstrated that nuclear DNA (nucDNA) imposed a selection on mitochondrial DNA (mtDNA) in human populations (Wei et al., 2019). These mitonuclear interactions contribute to phenotypic variation but also to health and disease conditions (Reynolds et al., 2020), as reported by studies on human admixed populations that showed suboptimal regulation of mtDNA replication when its components are encoded by nuclear and mtDNA genes with different ancestry (Zaidi and Makova, 2019).

However, the identification of nucDNA–mtDNA interactions meets the statistical and computational challenge to test a huge number of hypotheses: the routine methods often fail to find informative nucDNA–mtDNA combinations (Sackton and Hartl, 2016), even if the model is restricted to pairwise interactions only (Moore and Williams, 2009). Following the constant increase in population genomic datasets, the potential of machine learning (ML) tools has been realized, and it was demonstrated that they can outperform traditional approaches implemented by population genetics studies (Schrider and Kern, 2018).

During human evolution, the main selective pressures that anatomically modern humans had to face were linked to pathogen variability and load, dietary changes, and climatic conditions. In this study, we investigate mitonuclear genetic interactions in relation to the latter condition.

Most likely, metabolic adaptations to low temperatures occurred in several human populations when modern humans spread across Europe (Piazza et al., 1981; Wheeler, 1985; Sazzini et al., 2014; Quagliarello et al., 2017). The related adaptive traits have been achieved by modulation of thermogenesis and thermoregulation processes, which involve complex functional pathways, controlled by sympathetic signals of the hypothalamus produced in response to cold exposure, and in which mitochondria play a major role (Steegmann, 2007; Balloux et al., 2009). In fact, mitochondrial enzymatic activity is upregulated in skeletal muscle upon adaptation to increasing cold (Wakabayashi et al., 2017) and changes in mitochondrial architecture have been described to increase functionality in both skeletal muscle and brown adipose tissue (BAT) (Bal et al., 2017).

Here, we aim to identify statistically significant nucDNA–mtDNA interactions in human populations of European ancestry from different biogeographical and climatic areas by an ML approach. We analyzed a subset of 28 nuclear genes (Table 1) involved in cold adaptation and BAT metabolism and on all the mitochondrial genes.

**TABLE 1:** Number of single nucleotide variants (SNVs) in considered genes.

**Gene**	**Chromosome**	**Location**	**Number of SNVs**
**Mitochondrial DNA**			
*MT-ND3*	MT	NC_012920.1 (10059.10404)	77
MT-ATP6	MT	NC_012920.1 (8527.9207)	226
MT-ATP8	MT	NC_012920.1 (8366.8572)	73
MT-CO1	MT	NC_012920.1 (5904.7445)	319
MT-CO2	MT	NC_012920.1 (7586.8269)	152
MT-CO3	MT	NC_012920.1 (9207.9990)	182
MT-CYB	MT	NC_012920.1 (14747.15887)	326
MT-ND1	MT	NC_012920.1 (3307.4262)	218
MT-ND2	MT	NC_012920.1 (4470.5511)	236
MT-ND4	MT	NC_012920.1 (10760.12137)	291
MT-ND5	MT	NC_012920.1 (12337.14148)	408
MT-ND6	MT	NC_012920.1 (14149.14673)	128
MT-RNR1	MT	NC_012920.1 (648.1601)	118
**Nuclear DNA (GRCh38, hg38)**			
ADRA1A	11	NC_000008.11 (26738113.26870994)	7311
ADRB3	8	NC_000008.11 (37962990.37966599)	203
CIDEA	18	NC_000018.10 (12254361.12277595)	1,445
CREB1	2	NC_000002.12 (207529943.207605988)	4,353
DIO2	14	NC_000014.9 (80197526.80231057)	10,223
FTO	16	NC_000016.10 (53703963.54121941)	23,729
HOXC4	12	NC_000012.12 (54016888.54056030)	1,801
HOXA1	7	NC_000007.14 (27092993.27096000)	149
LIPE	19	NC_000019.10 (42401512.42427421)	1,485
LEP	7	NC_000007.14 (128241201.128257629)	863
LEPR	1	NC_000001.11 (65420652.65641559)	11,705
NRF1	7	NC_000007.14 (129611720.129757082)	6,949
NRIP1	21	NC_000021.9 (14961235.15065903)	753
PLIN1	15	NC_000015.10 (89664365.89679367)	865
PLIN2	9	NC_000009.12 (19108391.19127606)	2,751
PLIN3	19	NC_000019.10 (4838341.4867667)	2,141
PLIN5	19	NC_000019.10 (4522531.4535224)	999
PPARG	3	NC_000003.12 (12287368.12434344)	7,539
PPARGC1A	4	NC_000004.12 (23792021.24472905)	5581
PPARGC1B	5	NC_000005.10 (149730302.149857861)	7,307
PRDM16	1	NC_000001.11 (3069203.3438621)	28,237
PRKAR1A	17	NC_000017.11 (68413623.68551316)	1,869
PRKAR2A	3	NC_000003.12 (48744601.48847874)	4,663
PRKAR1B	7	NC_000007.14 (549185.727676)	14,603
PRKAR2B	7	NC_000007.14 (107044705.107161811)	6,691
UCP1	4	NC_000004.12 (140555770.140568961)	369
UCP2	11	NC_000011.10 (73974671.73983202)	517
UCP3	11	NC_000011.10 (74000277.74009237)	587

## Materials and Methods

### Data

**FIGURE 1:**
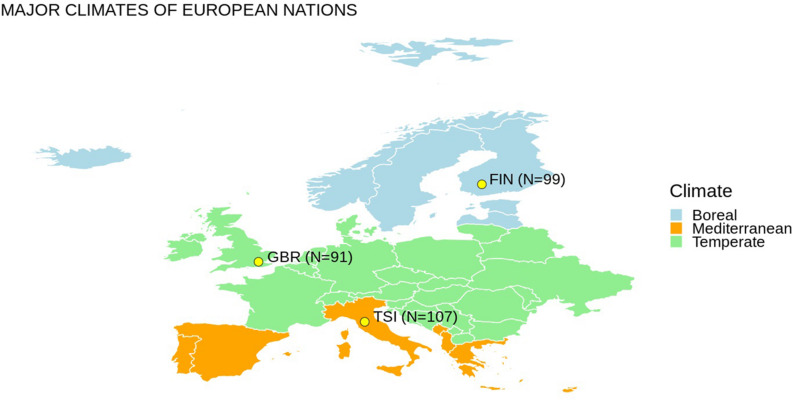
Europe map with populations considered in this study and an oversimplified representation of different climate regions. N indicates the number of individuals considered for each population.

We selected representative populations of European ancestry from different latitudes from the 1000 Genomes Project dataset (The 1000 Genomes Project Consortium, 2015). Genetic variants for 297 subjects from three populations (i.e., GBR – British in England and Scotland, FIN – Finnish in Finland, TSI – Tuscany in Italy) were extracted (Figure 1). We considered 13 mitochondrial genes and 28 nuclear genes (Table 1), which have been previously associated with cold adaptation (Sazzini et al., 2014). We selected only European populations for two main reasons: (1) they are characterized by limited genetic admixture; (2) they are representative of the western European genetic variability observable at different latitudes. Differences in climatic areas in Europe are not only attested by current data but appeared also in ancient times. For example, environmental temperature is one indicator of different climate zones, and time-series information was extracted for 2001–2010 from a 0.5° × 0.5° grid matrix assembled at the Climate Research Unit of the University of East Anglia as recently suggested (Key et al., 2018). By considering the geographic coordinates of each population, annual mean temperatures for FIN, GBR, and TSI are 5.7, 10.00, and 14.2°C, respectively. Concerning European ancient data, a recent paper (Singarayer and Valdes, 2010) described the global climate model for multiple snapshots from 120 kya to the present (for details, see^1^).

### Machine Learning Methods

We estimated relevant mtDNA–nucDNA interactions by identifying those combinations of mtDNA–nucDNA variants that manifest statistically significant association to the considered populations. In detail, they were formalized as features that received high importance scores for ML discrimination of the populations and made up classifiers with higher accuracy than those based on exclusively mitochondrial or nuclear genomes.

In the first step, we filtered out those single nucleotide variants (SNVs) in the considered genes that did not vary between all subjects. Table 1 represents the total number of remaining SNVs in these genes. Considering all the remaining combinations of mtDNA–nucDNA, SNVs would still pose a problem of huge dimensionality. That is, the simplest one of mitochondrial ATP8 and nuclear HOXA1 gives rise to 73×149 = 10.877 SNV pairs, while MT-ND5 and PRDM16 yield 408×28,237 = 11.520.696 SNV pairs. To further reduce the total number of considered combinations, we developed a computational procedure to match a score to a specific mitochondrial or nuclear gene, or an mtDNA–nucDNA gene pair.

By considering mitochondrial DNA only and by fixing a certain population as the reference one, we calculated the mean frequency $f_{G\,e\,n\,e\,N}^{r\,e\,f}\,\left(0/1\right)$ of each variant (0 or 1) for each gene. Then for each subject in each population and each gene N, we calculated a frequency score as the mean distance from the reference population by using the following equation:

$$f_{G\,e\,n\,e\,N}\,\left(0/1\right)=\frac{\sum_{a\,l\,l\,S\,N\,P\,s\,i\,n\,G\,e\,n\,e\,N}\left(1-f_{G\,e\,n\,e\,N}^{r\,e\,f}\,\left(0/1\right)\right)}{n\,u\,m\,S\,N\,P\,s\,i\,n\,G\,e\,n\,e\,N}$$

We followed the same approach for nuclear DNA variants, taking into account three variants (0|0, 0|1 or 1|0, 1|1), and for combinations of mtDNA–nucDNA variants, taking into account six combinations variants (0 + 0|0, 0 + 0|1 or 0 + 1|0, 0 + 1|1, 1 + 0|0, 1 + 0|1 or 1 + 1|0, 1 + 1|1). In result, the dimension of data for an individual was reduced to the number of mtDNA or nucDNA genes or their product.

The frequency scores were treated as features for the random forest (RF) algorithm to build and investigate binary classification models between each pair of populations. The implementation was taken from the Python package “scikit-learn” version 0.23; Python version 3.7. The number of decision trees was set to 500. Tenfold cross-validation was applied to test the effectiveness of the produced model, yielding the average accuracy over cross-validated models.

We thus performed a two-step classification experiment. The first one employed all constructed features assigning them with classification importance scores from the interval [0; 1]. Using the ranked feature lists, we performed serial RF experiments, increasing the number of features each time by one, until it made the whole list. The resulting accuracy plots are given in Supplementary Figure 1. Then, we determined the optimal classification, by balancing the accuracy score against the number of features. That is, if the classification accuracy for a smaller number of features was no worse than 1%, it took the preference.

**FIGURE 2:**
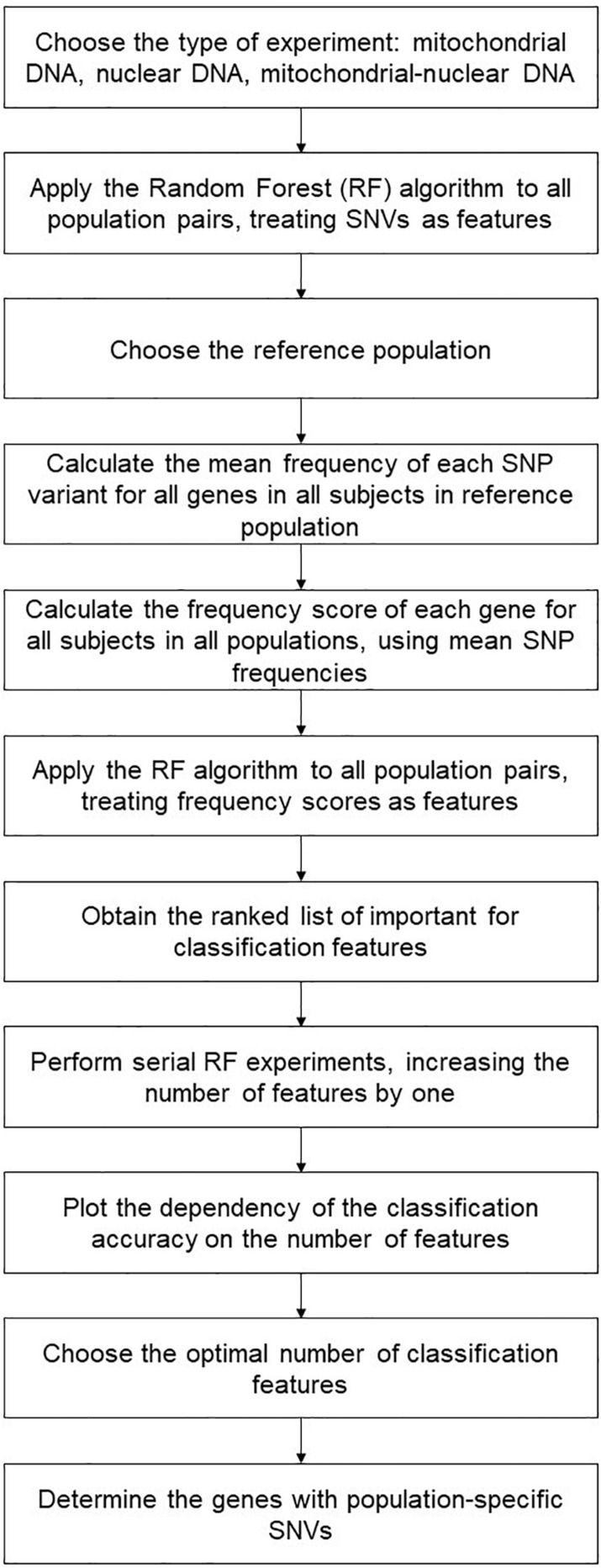
Main steps of the random forest approach.

We also defined genes and gene pairs with population-specific SNVs as those present in all resulting classification lists that were obtained for a given population (we termed them “population-specific genes”). For example, for the GBR population, it would be the intersection of the feature lists for the following classifiers: GBR (reference) vs. FIN (target), GBR (reference) vs. TSI (target), FIN (reference) vs. GBR (target), TSI (reference) vs. GBR (target). The main steps of the algorithm are presented in Figure 2.

For a single nuclear DNA gene (*FTO*) and all mitochondrial DNA genes, we also performed a random forest approach for SNVs, without choosing a reference population and by averaging over genes (we termed them “population-specific SNVs”). We treated SNV combinations (0 + 0|0, 0 + 0|1 or 0 + 1|0, 0 + 1|1, 1 + 0|0, 1 + 0|1 or 1 + 1|0, 1 + 1|1) as features for the random forest, and classification accuracy was calculated. A total of 100 SNV combinations with the highest classification importance scores for all experiments (GBR vs. FIN, GBR vs. TSI, FIN vs. TSI) were obtained. The population-specific SNV combinations were also defined as those present in all resulting features lists that were obtained for a given population.

### Population Structure Analysis

To explore patterns of population genetic structure among the three selected populations and to remove possible bias due to correlation between SNVs, the PLINK software v.1.9 (Chang et al., 2015) was used to prune out all the variants in linkage disequilibrium (LD). The pruning procedure consisted of selecting a window of 50 contiguous SNVs, calculate the LD between all SNV couples, and if the LD (*r*^2^) of a given couple was higher than 0.1, keep only one of the two SNVs, move the window forward of 10 SNVs and repeat the procedure.

The obtained pruned data-sets were used to perform population structure analyses commonly used in population genetics studies: discriminant analysis of principal components (DAPC) (Jombart et al., 2010) and the Fst statistic (Wright, 1949), which were performed for each nuclear gene and each mitochondrial gene separately.

DAPC describes genetic relationships among predefined groups of individuals minimizing the variance within the groups and optimizing the variance between the groups. To visualize the genetic distance among the three populations, we plotted DAPC results for the individuals in the coordinates defined by the first two principal components (PCs). The Euclidean distance between the centroids of the three populations was computed to quantify their genetic divergence.

Fst is a measure of population substructure and is most useful for examining the overall genetic divergence among subpopulations. Here, we computed the pairwise Fst test between all population pairs, so three Fst analyses were carried out for each gene. Then, to evaluate the significance of the observed Fst values, we performed 200 simulations, where individuals were randomly assigned to one of the three studied populations, and for each simulation, the Fst statistic was computed. At the end of this procedure, we were able to fit a probability density function for the Fst statistic and to compute an empirical *p*-value for the Fst value observed on the original data. A gene was considered discriminant for a given couple of populations if the empirical *p*-value was lower than 0.05. A gene was considered specific for a single population (for example FIN) if the empirical *p*-value was significant in the two Fst analyses involving the population itself (example FIN vs TSI and FIN vs GBR but not TSI vs GBR). A gene was considered specific for all the considered populations if the empirical p-value was significant in all the three Fst analyses.

Both the approaches were implemented with the R software (R Core Team, 2019) using the *adegenet* package (Jombart, 2008; Jombart and Ahmed, 2011) for the DAPC and the *hierfstat* package (Goudet, 2015) for the Fst statistic.

## Results

### Classifying Populations With Random Forest

We started with investigating the principle possibility of classifying the three considered populations of European ancestry (cf. Figure 1) with the ML random forest (RF) algorithm based on the SNV frequency scores for mtDNA genes, cold adaptation associated nucDNA genes, and their pair combinations (cf. Table 1). For each population pair, we performed two classification experiments, taking one or another as a reference and target. For example, GBR vs. FIN classification was made in two ways: GBR (reference) vs. FIN (target), FIN (reference) vs. GBR (target). Table 2 summarizes the accuracy of the resulting classification. The numbers of genes, used to achieve that accuracy, are reported in brackets. Note, that as expected, the table is unsymmetric, as these populations have different frequencies of SNVs variants, and yield different frequency score features, depending on which one is taken as reference.

**TABLE 2:** Classification accuracy for the considered populations.

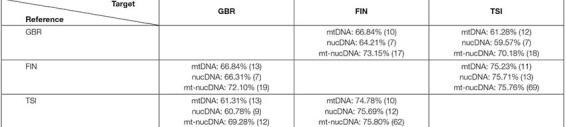

For all pairs of populations, the combinations of mitochondrial and nuclear DNA manifested better classification results than those based on mtDNA or nucDNA exclusively. In particular, the maximal accuracy increase was observed for the GBR-TSI classification. The FIN and TSI populations demonstrated superior classification accuracy in all cases, which is presumably the effect of their deeper genetic differences and geographic distance.

### Identifying Population-Specific Genes by ML

Next, we investigated the commonality between the features employed by different RF classifiers for each population to obtain the lists of genes, which invariably identify the population against the others. Such population-specific genes emerging for different experiment types (i.e., mtDNA, nucDNA, mtDNA–nucDNA, cf. Materials and Methods section) are listed in Table 3.

**TABLE 3:** Population-specific genes determined by the machine learning (ML) analysis.

**GBR**	**FIN**	**TSI**
**mtDNA**		
*MT-ATP6*, *MT-ND5*, *MT-CYB*, *MT-CO1*, *MT-CO3*, *MT-ND3*, *MT-ND2*, *MT-ND1*, *MT-ND6*, *MT-ND4*	*MT-ATP6*, *MT-ND5*, *MT-CYB*, *MT-CO1*, *MT-CO3*, *MT-ND3*, *MT-ND2*, *MT-ND1*, *MT-ND6*, *MT-ND4*	*MT-ATP6*, *MT-ND5*, *MT-CYB*, *MT-CO1*, *MT-CO3*, *MT-ND3*, *MT-ND2*, *MT-ND1*, *MT-ND6*, *MT-ND4*
**nucDNA**		
*PDRM16*, *LEPR*, *DIO2*	*PDRM16*, *LEPR*, *DIO2, PPARG*, *NRF1*	*PDRM16*, *LEPR*, *DIO2, UCP3*, *CIDEA*, *PLIN1*
**mtDNA-nucDNA**		
	*MT-CYB* + *FTO*, *MT-ND2* + *HOXA1 MT-CYB* + *PRDM16*, *MT-ND5* + *NRF1 MT-CYB* + *DIO2*, *MT-ND1* + *NRF1, MT-CYB* + *HOXA1*, *MT-ND3* + *NRF1 MT-ND1* + *HOXA1*, *MT-CYB* + *NRF1*	*MT-ATP6* + *PRDM16*, *MT-CYB* + *UCP2 MT-ND1* + *CIDEA*, *MT-ND3* + *DIO2 MT-RNR1* + *DIO2*, *MT-CO3* + *LEPR, MT-ATP6* + *UCP2*, *MT-ND2* + *CIDEA MT-RNR1* + *CIDEA*, *MT-ND4* + *DIO2*

All 10 mitochondrial population-specific genes were found to be common for the considered populations. Three nuclear population-specific genes (*PDRM16*, *LEPR*, and *DIO2*) were common as well; meanwhile, the other two genes (*PPPARG* and *NRF1*) resulted specific for the Finnish population, and three genes (*UCP3*, *CIDEA*, and *PLIN1*) turned out to be specific for the Italian population only.

We compared these results with those obtained by conventional methods commonly used in population genetics, such as DAPC and Fst.

Results from these two approaches are, in general, consistent with those that emerged from the ML approach for nuclear genes, except the *PLIN1* gene, which was identified as having specific SNVs for the Italian population by RF algorithm, but not discriminant for DAPC and Fst (Supplementary Figures 2–9 and Supplementary Table 1).

As to the mitochondrial genes, we performed only Fst analysis as DAPC is optimized for a large genomic dataset and the number of mtDNA variants in each gene was too low to give reliable results.

Considering Fst, we were able to well discriminate the Finnish from the other populations (Supplementary Figures 10–19). These results are concordant with those of the RF algorithm, where the 10 genes were classified as “population-specific genes” for all the three considered populations (Supplementary Table 2) suggesting that for mitochondrial genes, RF can better differentiate groups if compared to Fst.

Then, we proceeded to the mtDNA–nucDNA combinations identified by ML classifiers. A total of 10 mitochondrial–nuclear DNA combinations were found to be population specific for the Finnish and Italians. Table 3 shows that for FIN, *FTO*, and *HOXA1* genes were not population specific alone but only in combination with mitochondrial genes. For TSI, the *UCP2* gene did not have population-specific SNVs alone but had population-specific SNV combinations with mitochondrial genes. No such population-specific combinations of genes were observed for the British population.

### Classification Power of Mitochondrial–Nuclear DNA Combinations

The above results indicate that population classification accuracy can be improved by combining mitochondrial and nuclear gene variants into the ML analysis. We then addressed the question on whether the classification power for specific genes was improved when they get paired. For this purpose, we compared classification importance scores for the genes and their pair combinations in RF population classifiers that use (i) only mitochondrial DNA, (ii) only nuclear DNA, and (iii) combinations of mitochondrial and nuclear DNA genes.

The two most interesting cases are reported in Table 4: (1) mtDNA—nucDNA combinations that showed greater importance scores than mtDNA and nucDNA genes did separately and (2) mtDNA–nucDNA combinations that were recruited in population classifiers, while at least one of the single genes was not. For the pair of populations GBR vs. TSI, we obtained four combinations of mitochondrial and nuclear DNA, which showed an increased importance score when brought together. We have also found some combinations of genes that are useful in the classification of FIN vs. TSI, while single nuclear DNA or mitochondrial DNA genes were not even selected for classification.

**TABLE 4:** Genes that increase their classification power in combination.

**Reference population**	**Target population**	**Increasing classification importance when in combination**	**Classifying populations only when in combination**
GBR	FIN		*MT-ND3* + *NRF1 MT-ATP8* + *FTO*
GBR	TSI	*MT-CO3* + *LEPR MT-ATP6* + *PRDM16 MT-RNR1* + *CIDEA MT-RNR1* + *DIO2*	*MT-ND3* + *DIO2*
FIN	GBR		*MT-ND3* + *NRF1*
FIN	TSI		*MT-CO2* + *PLIN2 MT-CO2* + *LEPR MT-ATP8* + *PRDM16 MT-ND3* + *DIO2 MT-RNR1* + *PRDM16 MT-ND3* + *FTO MT-ND3* + *PRDM16 MT-RNR1* + *CIDEA MT-CO2* + *UCP3 MT-ATP8* + *LEPR*
TSI	GBR	*MT-RNR1* + *DIO2 MT-RNR1* + *CIDEA*	*MT-ND3* + *DIO2*
TSI	FIN		*MT-ATP8* + *PRDM16 MT-ND3* + *DIO2 MT-RNR1* + *PRDM16 MT-ND3* + *FTO MT-ND3* + *NRF1 MT-RNR1* + *LEPR MT-RNR1* + *FTO MT-ATP8* + *LEPR MT-ATP8* + *FTO MT-ATP8* + *DIO2 MT-RNR1* + *UCP3 MT-RNR1* + *UCP2*

Last, Table 5 summarizes all the genes originally chosen as associated with cold adaptation, and reports which genes were pinpointed to likely play a major population-specific role alone, and which instead showed statistical epistasis with mtDNA. The first class was defined based on the list of nuclear genes from Table 3. The second class was based on the results of Table 4 so that the statistical epistasis was noted when a nuclear gene becomes a classifying feature or increases its classification importance being paired with a mitochondrial gene.

**TABLE 5:** List of nuclear genes with their roles in classifying populations.

**Nuclear gene**	**Gene is important alone**	**Gene is important with mtDNA**
*ADRA1A*	−	−
*ADRB3*	−	−
*CIDEA*	TSI	TSI
*CREB1*	−	−
*DIO2*	all populations	TSI
*FTO*	−	FIN
*HOXC4*	−	−
*HOXA1*	−	−
*LIPE*	−	−
*LEP*	−	−
*LEPR*	all populations	TSI
*NRF1*	FIN	FIN
*NRIP1*	−	−
*PLIN1*	TSI	−
*PLIN2*	−	FIN vs. TSI
*PLIN3*	−	−
*PLIN5*	−	−
*PPARG*	FIN	−
*PPARGC1A*	−	−
*PPARGC1B*	−	−
*PRDM16*	all populations	TSI
*PRKAR1A*	−	−
*PRKAR2A*	−	−
*PRKAR1B*	−	−
*PRKAR2B*	−	−
*UCP1*	−	−
*UCP2*	−	FIN vs. TSI
*UCP3*	TSI	FIN vs. TSI

*The middle column is based on Table 3. The right column is based on Table 4. Importance is marked either for classification of specific populations [“Finnish in Finland (FIN) vs. Tuscany in Italy (TSI)”], or for classification of a population against the other two (“TSI”), or for classification of every population against the other two (“all populations”).*

### Identification of Population-Specific Variants

As the number of SNV pairs in all genes was enormous, we considered only the *FTO* nuclear gene in combination with all mitochondrial genes. We applied the RF model, using SNV combinations variants as an input and performed binary classification experiments for all population pairs. As for the previous method, the classification accuracy was higher for the combinations in comparison to only mitochondrial or nuclear DNA (Table 6). As a result, we obtained 100 SNV combinations with the highest values of classification importance. Next, we intersected the obtained lists of the most important SNV combinations for all binary classification experiments (GBR vs. FIN, GBR vs. TSI, and FIN vs. TSI).

**TABLE 6:** Classification accuracy for the SNV combinations of FTO nuclear gene with mitochondrial genes for all considered populations.

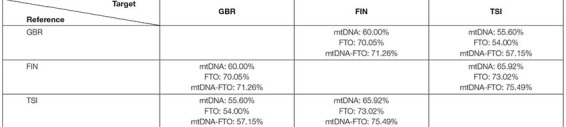

There were no common SNV combinations, important for all populations. We also intersected lists for certain populations and found three specific combinations for FIN population SNV pairs: 9066 (*MT-ATP6*) with rs12933928 (*FTO*), 12618 (*MT-ND5*) with rs12933928 (*FTO*), and 7124 (*MT-CO1*) with rs12933928 (*FTO*). SNV rs12933928 resulted specific for the FIN population in terms of nuclear DNA experiment, and SNV rs2388451 was specific for TSI.

## Discussion

Mitonuclear interaction represents a fundamental biological process for complex life, and such intergenomic interaction has been described as undergoing an intense selection to maintain the integrity of mitochondria itself. It has been also demonstrated that mitonuclear gene interactions modulate the expression of phenotypic traits at different timescales and during life course (Wolff et al., 2014).

With such a premise, here we applied a method of ML to investigate and integrate genomic information coming from both nuclear and mitochondrial DNA. To reduce the huge number of possible combinations and keep the focus on one biological issue, we compared genetic variability patterns among human populations of European ancestry, and we selected 28 nuclear genes (Table 1) based on their biological function in processes related to cold adaptation, such as BAT formation. We decided to include the genes based on their functions even if not all of the evidence of genetic loci involved in cold response were reported. This is because we cannot exclude that a combination of nuclear and mitochondrial variants plays a role even if the single variant does not.

It is widely described that climate exerted a major role in shaping populations’ genomic diversity by impacting on several human traits, such as body size, skin pigmentation, energy expenditure, and nutrient metabolism (Hancock et al., 2011; Sturm and Duffy, 2012; Raj et al., 2013; Fumagalli et al., 2015; Quagliarello et al., 2017; Sazzini et al., 2020). At the same time, the mitochondria are crucial for heat production in many organisms and its metabolic role in ATP-production (as well as for a variety of other cellular functions, such as survival/cell death, differentiation, redox and inflammation regulation, and numerous metabolic processes). Along these lines, we selected three European populations from different latitudes (FIN, GBR, and TSI) and analyzed the available data from the 1000 Genomes project.

The first results showed that the accuracy for classification always increased when we looked at the combination of mtDNA-nucDNA, indicating a potential functional role of the combination in shaping the genetic diversity of these three populations.

Comparing ML to a combination of DAPC and Fst-based approaches aimed at exploring population genetic structure (i.e., methods widely used in population genetics studies), we showed a perfect match regarding the results of DAPC and Fst analyses and those pointed out by the ML method for nuclear genes. Conversely, for mtDNA genes, RF effectively increased the predictive power when compared to Fst, whereas single genes may be unable to discriminate populations.

DAPC and Fst analyses identified only *MT-ND5* and *MT-ND6* as population specific, while ML identified 10 genes (Table 3). Among them, *MT-ND3* and *MT-ATP6* were identified in line with a study on mtDNA of populations belonging to different climate areas, which demonstrated that variants are located in *MT-ND3*, and *MT-ATP6* showed a significant correlation with minimum temperatures (Balloux et al., 2009). Moreover, ML is likely to grasp differences in haplogroup compositions. Populations of European ancestry have been considered relatively homogeneous in terms of mtDNA variability; however, published data showed that haplogroups are largely shared between these populations, but their frequencies are appreciably different in the three considered populations (Underhill and Kivisild, 2007; Pinhasi et al., 2012). For instance, FIN showed a higher frequency of haplogroup U than GBR or TSI. The differences identified in Table 3 can be explained by haplogroup variability among populations and by considering the mutations that characterized haplogroups. In fact, mtDNA haplogroup U is characterized by mutations in positions 11,467, 12,308, and 12,372, and two of them are indeed located in the *MT-ND4* and *MT-ND5* genes. Haplogroup H is instead characterized by mutations in positions 73, 11,719, 2,706, and 7,028, and two of them are indeed located in the *MT-ND4* and *MT-CO1* genes. Haplogroup J is characterized by mutations in positions 11,251, 15,452, 16,126 + 295, 489, 10,398, 12,612, 13,708, and 16,069, which are located in *MT-ND4*, *MT-CYB*, *MT-ND3*, and *MT-ND5*, and haplogroup T is characterized by 11,251, 15,452, 16,126 + 709, 1,888, 4,917, 8,697, 10,463, 13,368, 14,905, 15,607, 15,928, and 16,294 mutated positions located in the *MT-ND4*, *MT-CYB*, *MT-ND2*, *MT-ATP6*, and *MT-ND5* genes.

Thus, it is interesting to note that ML can identify differences in terms of haplogroup distribution among the three populations analyzed that are only marginally identified by classical methods, such as DAPC and Fst analyses.

DAPC is well suited for identifying the genetic structure between two or more populations by finding linear combinations of alleles that enable the distinction between clusters in an optimal way, and by focusing on the inter-group variability and disregarding the intra-group one. However, its main limitations concerning statistical epistasis are (i) the unwanted sensitivity to associations between variables (that is expected to be present under epistasis hypothesis) and (ii) the ambiguity in determining the importance of the found multivariate principal components for the discrimination between the groups (Jombart et al., 2010). In particular, it confined the analysis to interrogating specific genes’ ability to discriminate between populations.

On the contrary, RF is free from these flaws: building several decision trees and combining them together increase the accuracy of the result. Moreover, the features are assigned with importance score that allows for the straightforward interpretation and comparison of different gene contributions to the performance of a multivariate classifier. Besides, the use of several trees reduces the risk of overfitting. The main limitation of an RF approach is that many trees can make the algorithm slow and computationally inefficient, which made us introduce aggregate gene SNV frequency scores instead of using all SNVs.

In this study, we thus identified population-specific combinations of nuclear and mitochondrial genes.

For TSI, the nuclear gene *UCP2* was the only gene that is not population specific alone but in combination with *MT-CYB* and *MT-ATP6* genes. For GBR, no combinations of nuclear and mitochondrial genes have been identified likely because GBR represents an intermediate population in terms of latitude. For FIN, the nuclear genes *HOXA1* and *FTO* turned out to be population specific only in combination with mitochondrial genes (*MT-ND1*, *MT-ND2*, and *MT-CYB*).

*UCP2* is expressed in many cell types, such as white adipose tissue and pancreatic beta cells, but its functions are still controversial (Diano and Horvath, 2012). Recent data showed that both fatty acids and ROS activate *UCP2* to buffer overproduction of ROS and allow efficient mitochondrial energy production (Andrews, 2010). It can be hypothesized that *UCP2* works to reduce ROS produced in population with warm climates to counteract the damage of ROS production. In fact, it has been described that different nuclear–mtDNA combinations influence organelle oxidant production in a population-specific way (Brown et al., 2020). American individuals of African ancestry, whose ancestry is related to geographical areas characterized by hot climates, have a more “economical” mitochondrial transport that produces the same level of ATP having a lower oxygen consumption, but at the same time producing more DNA damage (Krzywanski et al., 2016). Further studies are needed to accurately prove this hypothesis.

*HOXA1* belongs to the HOX gene family. These loci play a role in adult processes, such as embryo implantation, hematopoiesis, and endothelial differentiation.

Genetic mutations in *HOXA1* in different genetically isolated populations from Saudi Arabia and Turkey were reported (Tischfield et al., 2005). Individuals with these mutations are characterized by facial anomalies, but also by vascular malformations of the internal carotid arteries and cardiac outflow tract.

The cardiovascular system is a crucial player in the cold adaptation as much data suggested. Exposure to cold temperatures causes rises in vasomotor tone, hemodynamic parameters, platelet aggregability, and other hematological and endothelial parameters (Makinen, 2010). It is to mention that the rates of coronary events increase during cold periods and especially in a warm climate. It was also showed that the populations from cold regions (i.e., Northern Sweden, North Karelia, and Kuopio) showed little change in coronary event rates with changes in temperatures (Barnett, 2005). A possible explanation is that some populations, such as FIN, carry genetic variants (or combinations of them as in the case of *HOXA1*) that confer an advantage to cope with cold climates at the vascular level.

Moreover, the *FTO* gene emerged as the only gene that enables one to distinguish populations only when considered in combinations with certain mtDNA genes (Table 5).

*FTO* is the first obesity-susceptibility gene identified in genome-wide association studies, and it was identified in many different human groups (Loos and Yeo, 2014). Different patterns of *FTO* variation among populations have been described, and its relevant variants are found to be substantially less prevalent in populations with non-European ancestry (Loos and Yeo, 2014).

Its role in thermogenesis and its interaction with the mitochondria have been recently described. The *FTO* allele associated with obesity represses mitochondrial thermogenesis in adipocyte precursor cells in a tissue-autonomous manner (Claussnitzer et al., 2015). Moreover, *FTO* affects mitochondrial content and fat metabolism (Kang et al., 2018). The interconnection between *FTO* and mitochondria has been described as *FTO* downregulation suppressed mitochondria biogenesis and energy production, resulting in decreased mitochondria mass and mtDNA content (Kang et al., 2018). Moreover, recently Dunham-Snary and colleagues using Mitochondrial-Nuclear eXchange mice demonstrated that different mitochondrial–nuclear genome combinations influence metabolism, adiposity, and gene expression (Dunham-Snary et al., 2018). Furthermore, combinations of mtDNA and nucDNA have been associated with different metabolic traits, such as adipose measures (Kraja et al., 2019).

All these data support the recent biological evidence that highlights the possible interconnection between genetic variability of *FTO* and mtDNA.

We identified a single variant (rs12933928) located in the *FTO* gene that interacts with several mitochondrial variants and that characterized the Finnish population.

Single nucleotide variant rs12933928 is located at the end of the *FTO* locus far from the body mass index (BMI)-associated *FTO* intron 1 region. This variant has been shown to increase the risk of the degenerative disk disease and melanoma independent of obesity (Li et al., 2013; Lao et al., 2014; Kalo et al., 2020). Melanoma risk in Europeans is associated with a lighter skin pigmentation that has been proposed to be beneficial in northern latitudes to sustain vitamin D3 production in low-ultraviolet environments (Jablonski and Chaplin, 2010; the GenoMEL Consortium, 2013; Key et al., 2016).

Even if no functional data are present in the literature, it is interesting to note that mitochondria are implicated in the biosynthesis of melanin in melanocytes required to create the pigmentation in response to UV light (Rosania, 2005; Sreedhar et al., 2020). Moreover, it has been reported that melanoma skin cancer cells also generate a high level of reactive oxygen species (ROS) in their mitochondria, suggesting a hypothetical link between the identified signals in nuclear and mitochondrial genomes (Boulton and Birch-Machin, 2015).

In conclusion, we are aware of the limitations and strengths of this study.

The main limitations are:

(1) The fact that we identified statistical interactions at the genetic level, which need a functional validation using biological models (*in vivo* or *in vitro*);
(2) The absence of phenotypic data to test direct associations (mtDNA–nucDNA combination vs. potentially adaptive phenotypic traits);
(3) We cannot distinguish if the interactions identified may be due to migration/admixture patterns and the peculiar genetic history of the populations analyzed or to a potential selective advantage of having some combinations of nuclear and mitochondrial variants. Further studies are needed in this direction.

However, recent data support the fact that mitonuclear interactions are consistent with our understanding of the demographic history of human populations, stressing the absence of selective pressures to maintain mitonuclear combinations (Sloan et al., 2015). Despite that and irrespectively to the evolutionary forces that acted on these combinations, their impact on human health seems to be relevant (Kenney et al., 2014; Mottis et al., 2019; Zaidi and Makova, 2019; Rand and Mossman, 2020) and a further effort to find methods able to estimate the strength of selection on mitonuclear combination are needed.

The main strengths and novelty of this study are twofold (both methodological and biological):

• The method seems to be useful to detect patterns of genetic structure among human groups and to grasp different dimensions of the genetic variability that characterize human populations. It may be applied to single genes and combinations of genes. It is always concordant with the results of Fst and DAPC analyses, and in certain cases, it reported more informative genes with good accuracy (for example, for mtDNA). The use of ML was applied to describe genetic variation among human populations from different latitudes, but can be potentially applied to many other case studies of biological interest.
• The analysis identified nuclear genes that likely play a relevant biological role in association with a mitochondrial gene and that are population specific (such as *FTO*). The rationale used to select the analyzed genes supports the probability that these interactions may play a role in cold adaptation and related traits and gives suggestions for new target genes to be further investigated by *in vivo* and *in vitro* studies.

## Data Availability Statement

Publicly available datasets were analyzed in this study. This data can be found here: https://www.internationalgenome.org/category/phase-3/.

## Ethics Statement

The studies involving human participants were reviewed and approved by the 1000 Genomes Project Steering Committee. The patients/participants provided their written informed consent to participate in this study.

## Author Contributions

CG, MI, CF, and PG involved in the study design. AK, MI, VI, and SJ set up the mathematical models and performed analysis. CG, MS, and CF performed literature searching and biological interpretation. AK and CG wrote the first draft and all authors were involved in reviewing, and editing. All authors are responsible of the final contents and have read and approved the manuscript.

## Conflict of Interest

The authors declare that the research was conducted in the absence of any commercial or financial relationships that could be construed as a potential conflict of interest.

## Footnotes

1 https://www.paleo.bristol.ac.uk/ummodel/data/bbc_all_03/standard_new_html/bbc_all_03.html
