# Supplementary material for: Investigating Mitonuclear Genetic Interactions Through Machine Learning: A Case Study on Cold Adaptation Genes in Human Populations From Different European Climate Regions
Source: Front Physiol. 2020 Nov 11;11:575968. doi: 10.3389/fphys.2020.575968 (PMC7686538; doi:10.3389/fphys.2020.575968)
Supplement: Supplementary file 1 [file Table_1.DOCX]

**SUPPLEMENTARY FIGURES**


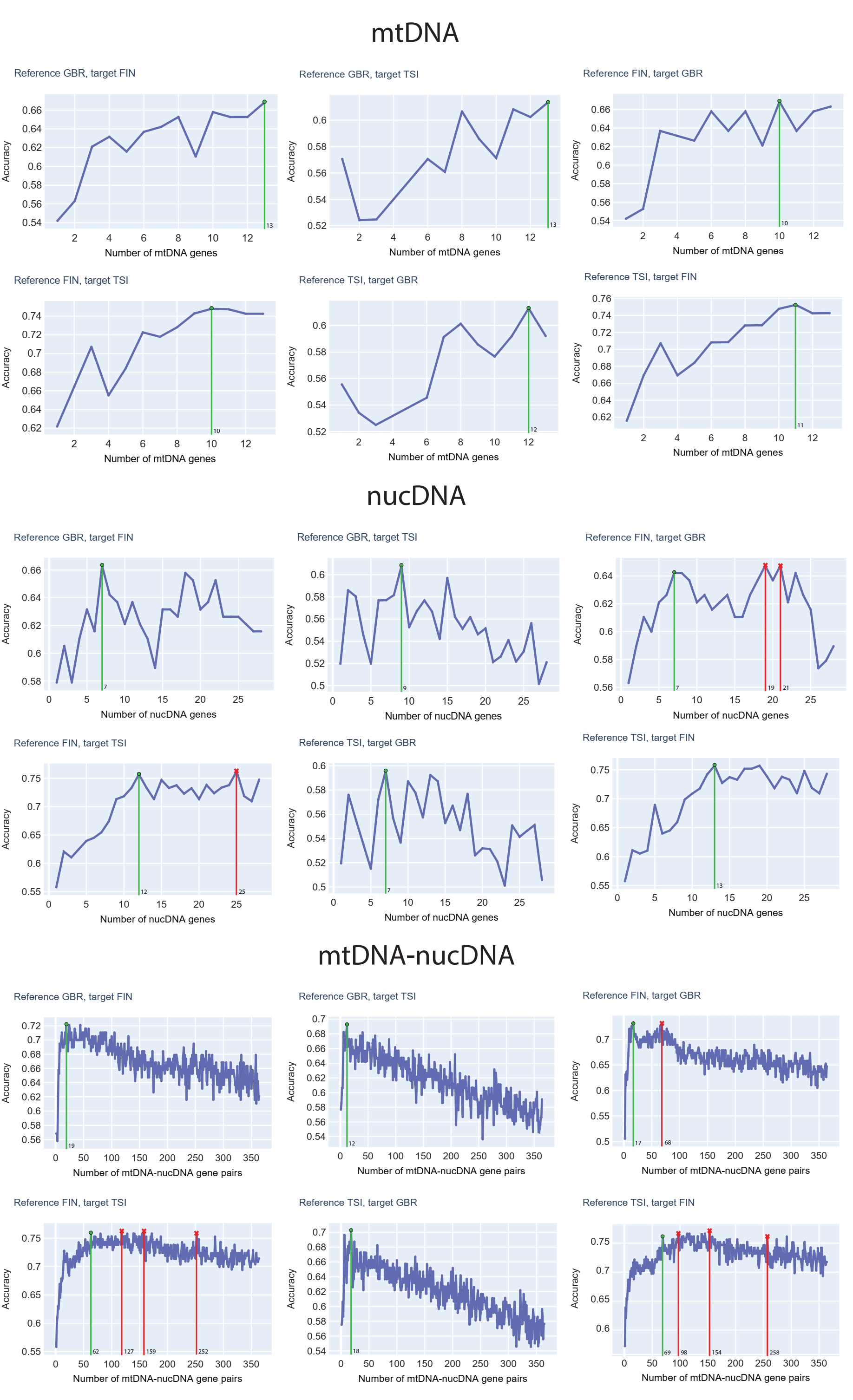


Figure S1: Dependence of classification accuracy on the number of features for 3 types of Random Forest experiments: mtDNA, nucDNA, mtDNA-nucDNA pairs. Green points correspond to optimal accuracy scores, red crosses – to rejected accuracy scores.


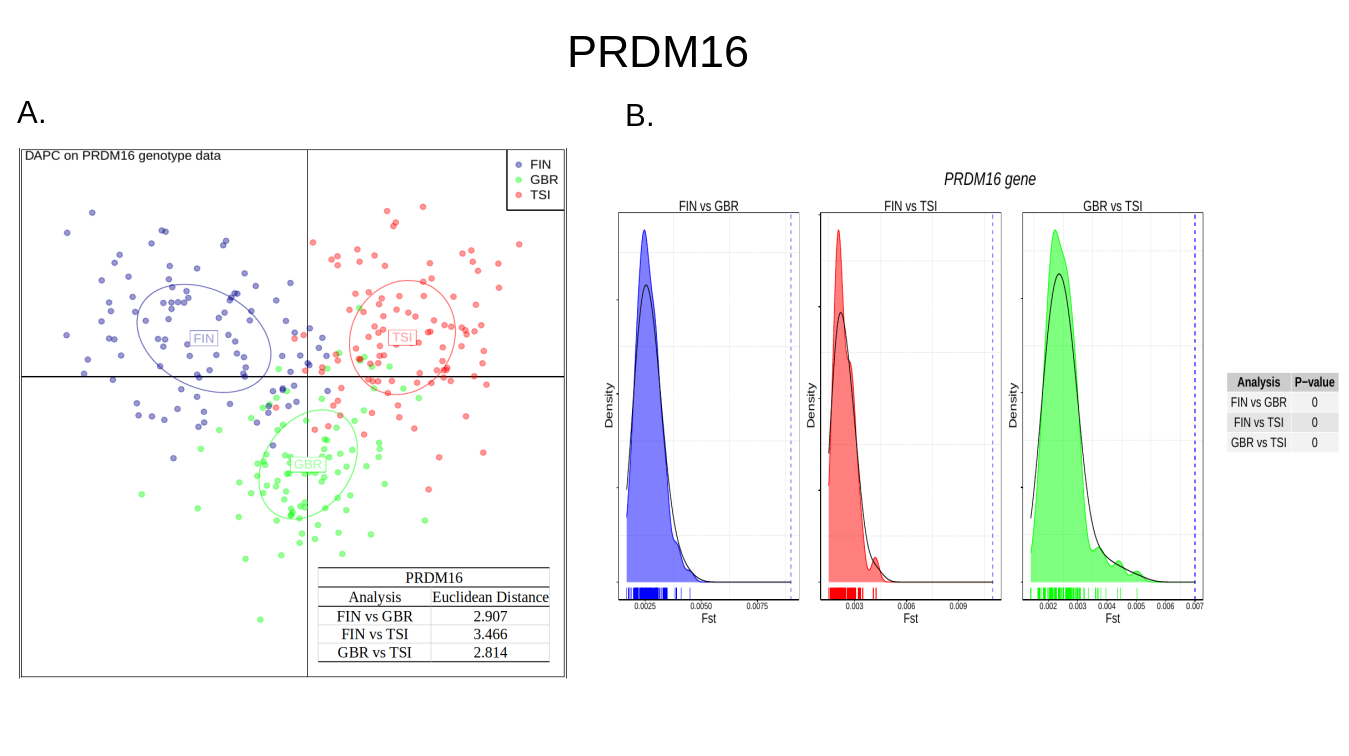


Figure S2: Genetic distance for PRDM16 gene considering Finnish (FIN), British (GBR) and Toscani (TSI). DAPC was calculated considering FIN, GBR and TSI (A). FST was calculated considering each populations couple. Dashed blue line indicates the Fst value observed on the original data (B).


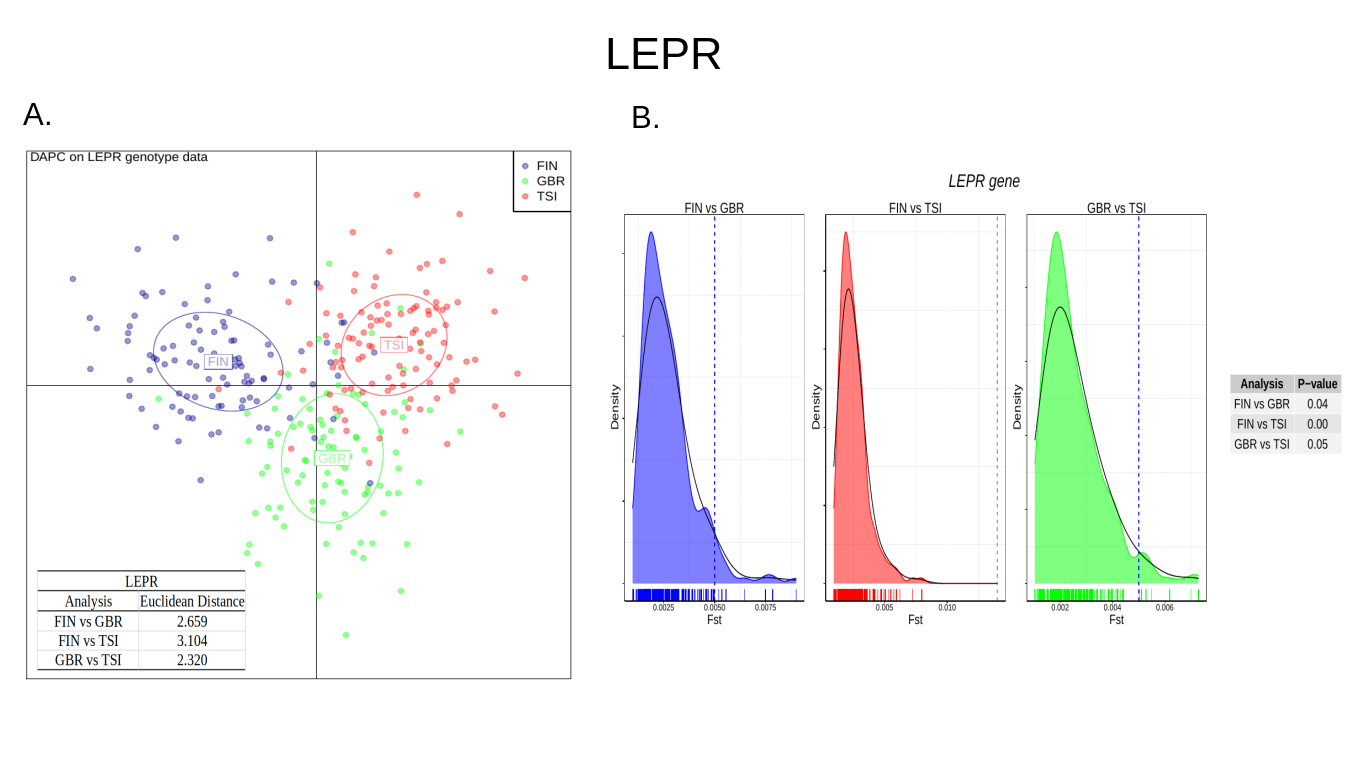


Figure S3: Genetic distance for LEPR gene considering Finnish (FIN), British (GBR) and Toscani (TSI). DAPC was calculated considering FIN, GBR and TSI (A). FST was calculated considering each populations couple. Dashed blue line indicates the Fst value observed on the original data (B).


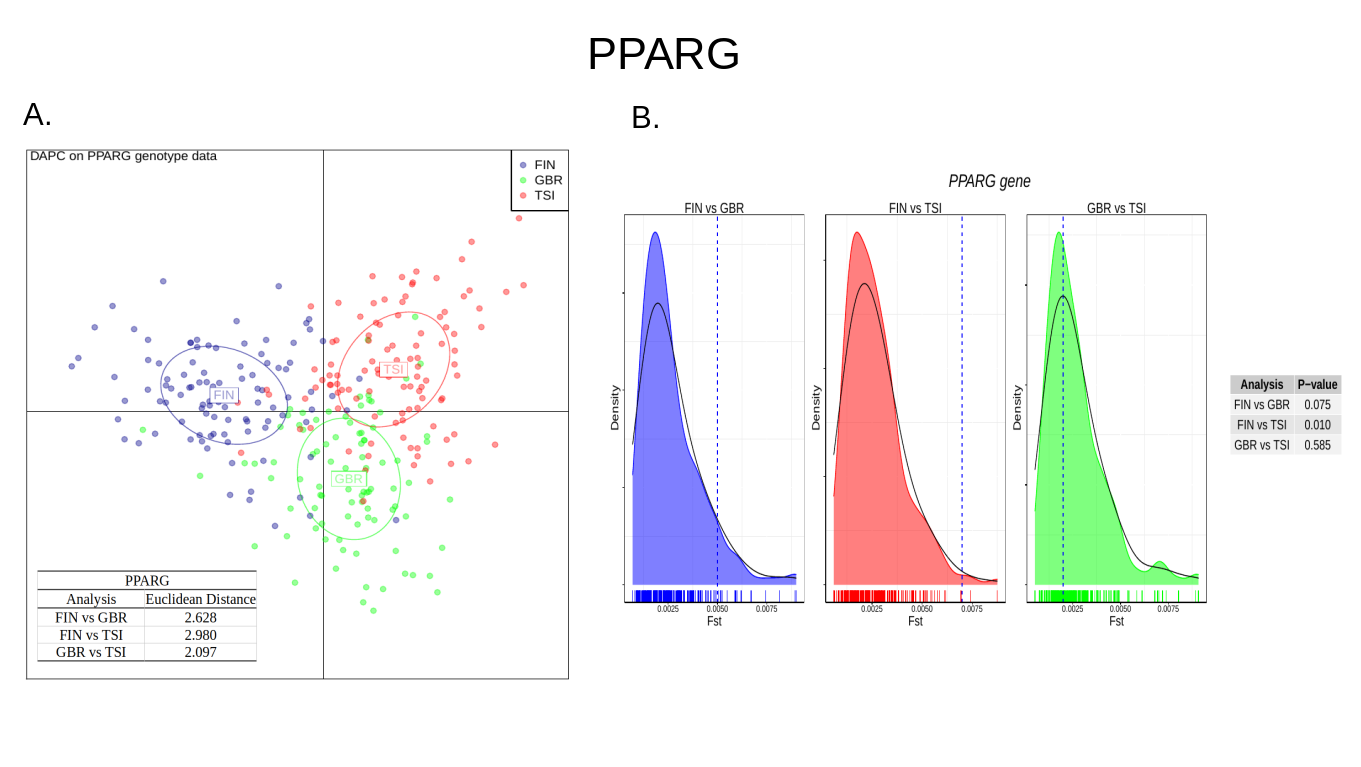


Figure S4: Genetic distance for PPARG gene considering Finnish (FIN), British (GBR) and Toscani (TSI). DAPC was calculated considering FIN, GBR and TSI (A). FST was calculated considering each populations couple. Dashed blue line indicates the Fst value observed on the original data (B)


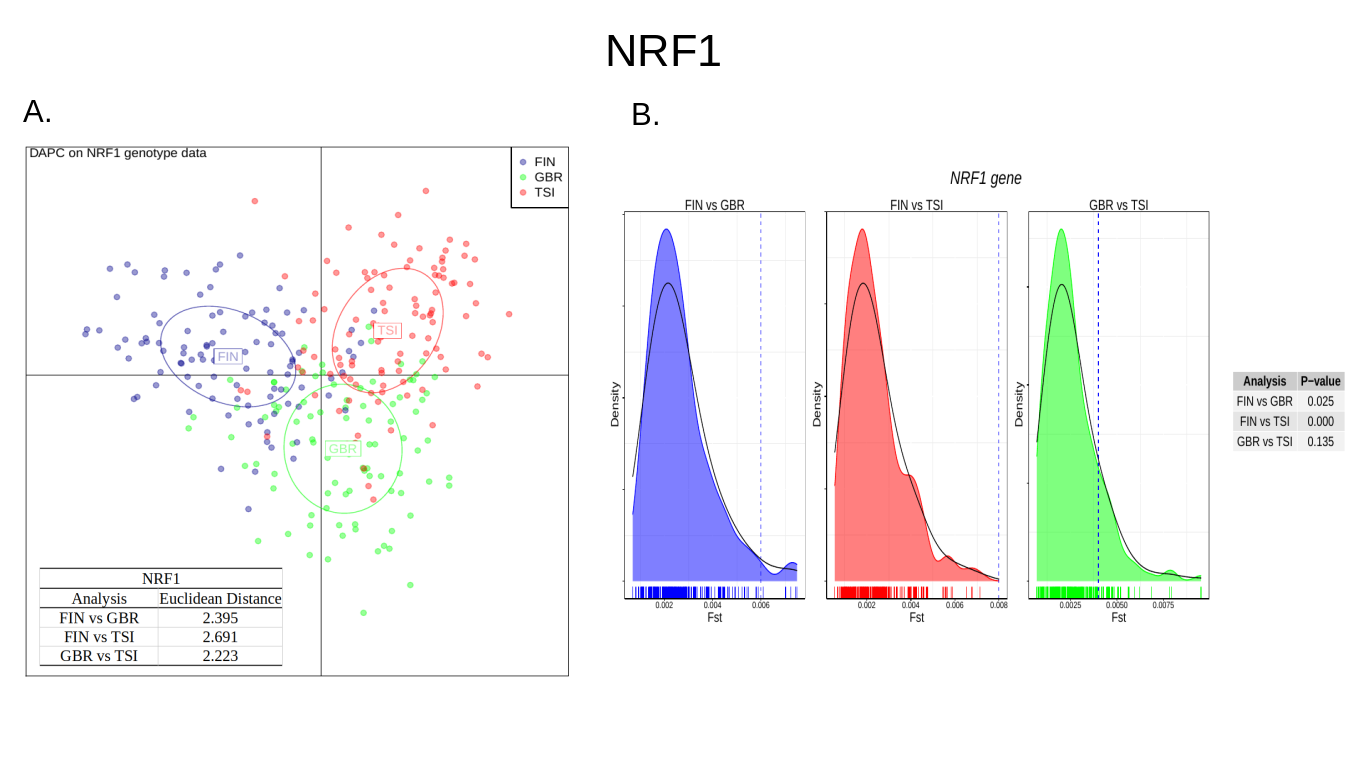


Figure S5: Genetic distance for NRF1 gene considering Finnish (FIN), British (GBR) and Toscani (TSI). DAPC was calculated considering FIN, GBR and TSI (A). FST was calculated considering each populations couple. Dashed blue line indicates the Fst value observed on the original data (B).


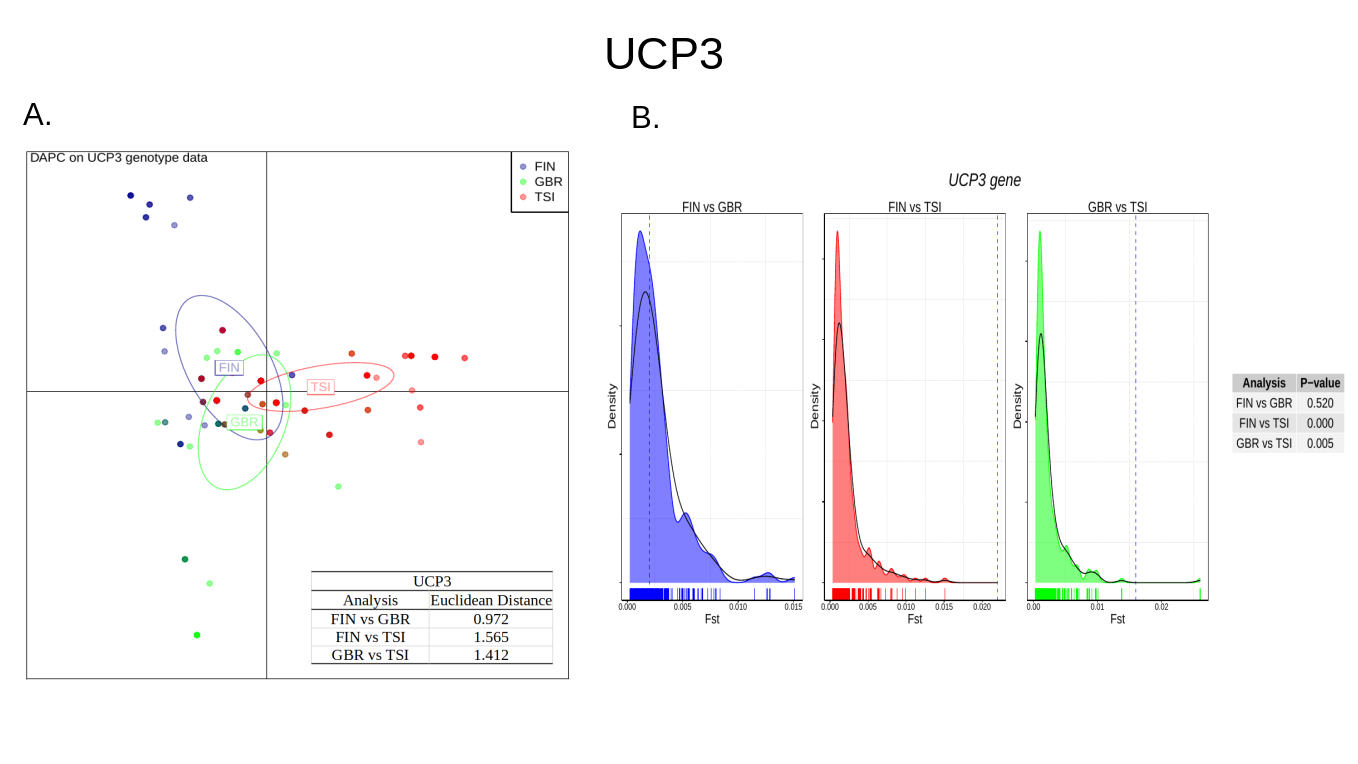


Figure S6: Genetic distance for UCP3 gene considering Finnish (FIN), British (GBR) and Toscani (TSI). DAPC was calculated considering FIN, GBR and TSI (A). FST was calculated considering each populations couple. Dashed blue line indicates the Fst value observed on the original data (B).


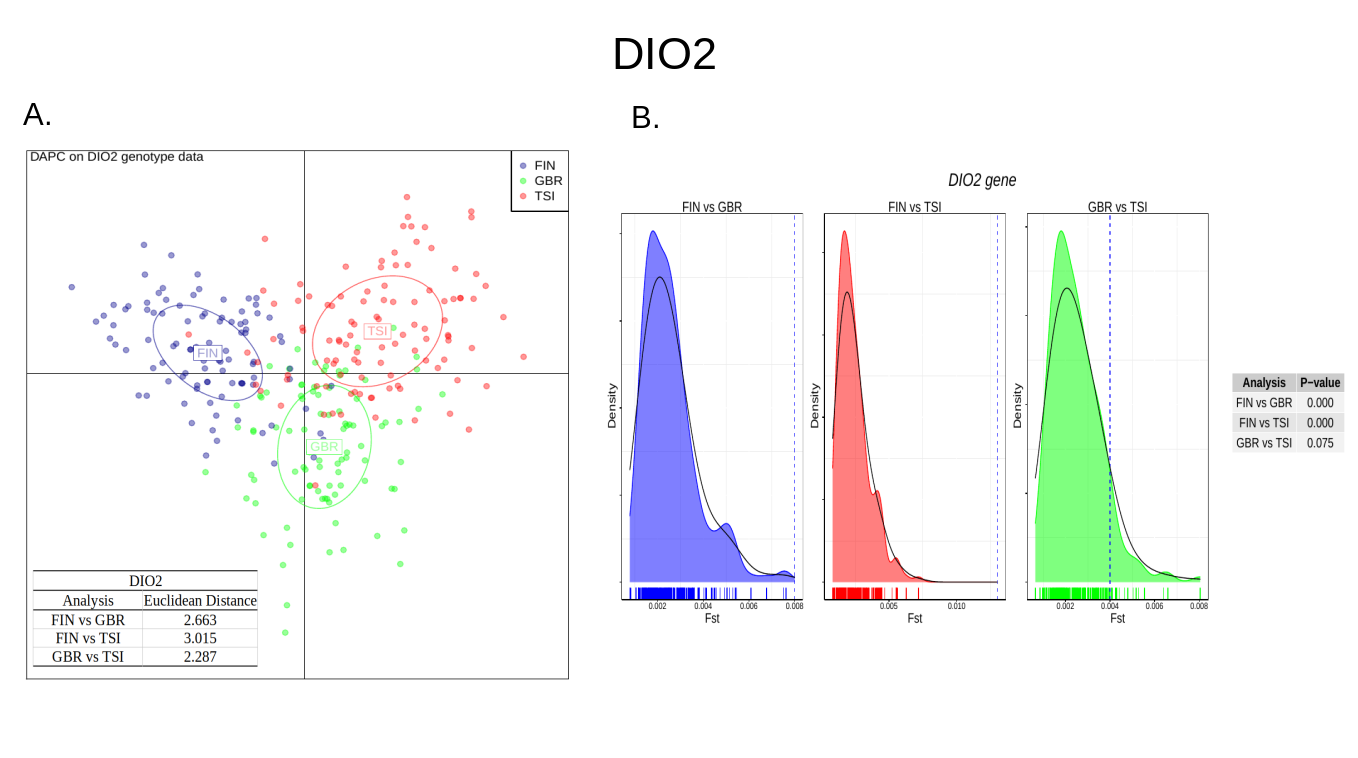


Figure S7: Genetic distance for DIO2 gene considering Finnish (FIN), British (GBR) and Toscani (TSI). DAPC was calculated considering FIN, GBR and TSI (A). FST was calculated considering each populations couple. Dashed blue line indicates the Fst value observed on the original data (B).


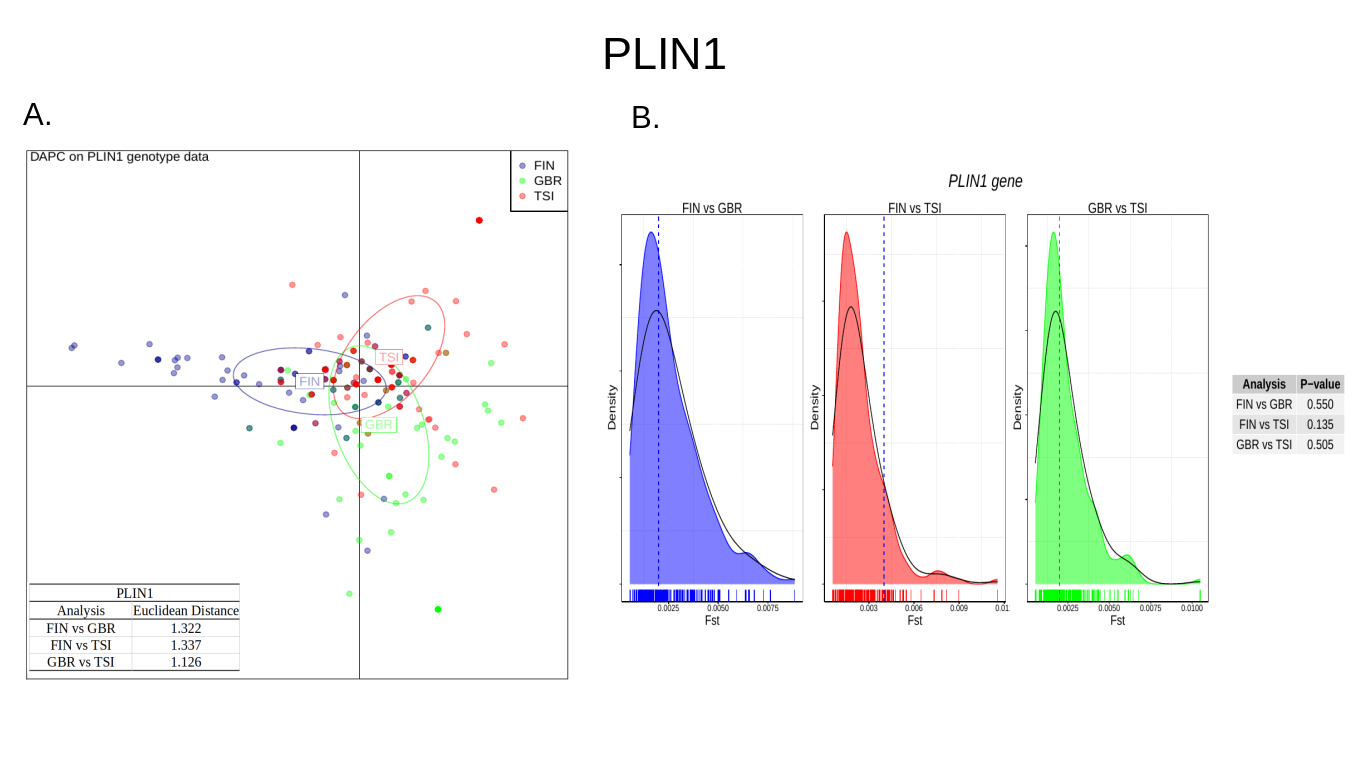


Figure S8: Genetic distance for PLIN1 gene considering Finnish (FIN), British (GBR) and Toscani (TSI). DAPC was calculated considering FIN, GBR and TSI (A). FST was calculated considering each populations couple. Dashed blue line indicates the Fst value observed on the original data (B).


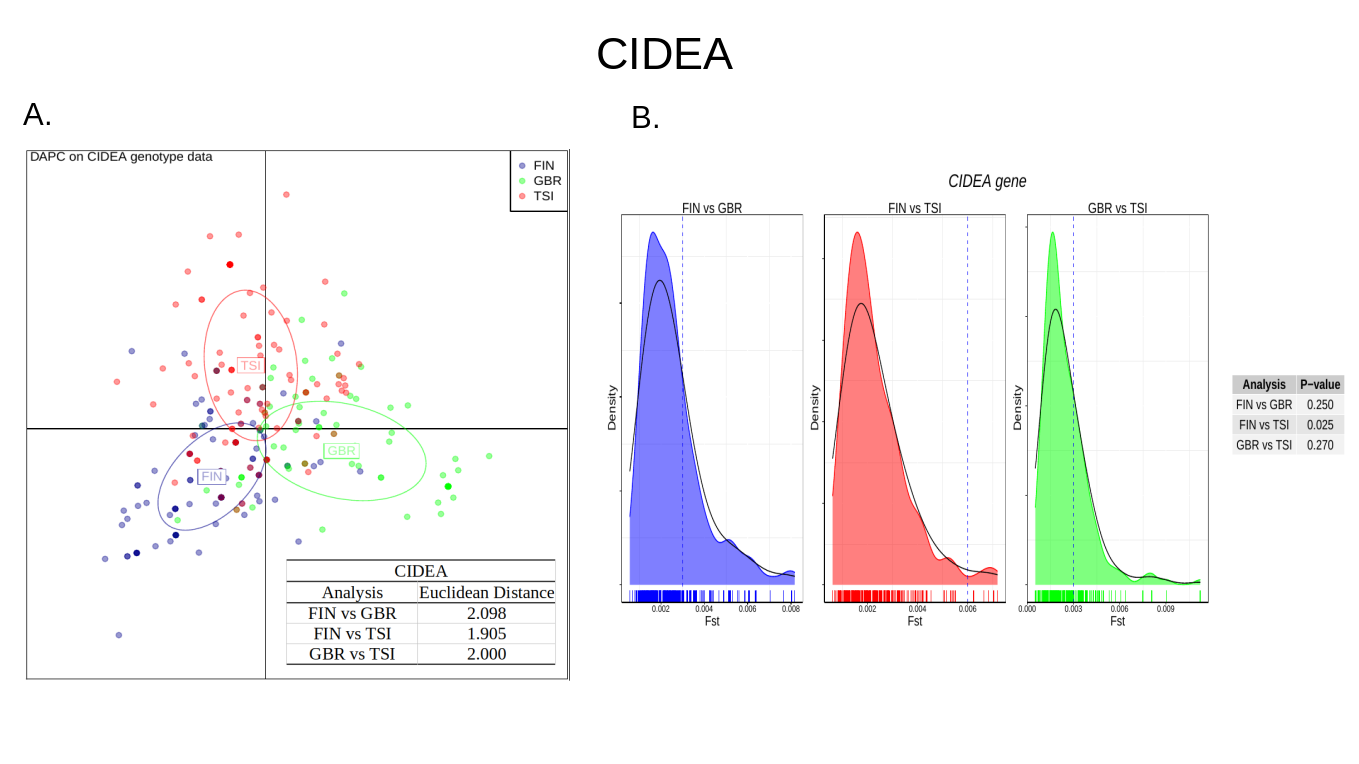


Figure S9: Genetic distance for CIDEA gene considering Finnish (FIN), British (GBR) and Toscani (TSI). DAPC was calculated considering FIN, GBR and TSI (A). FST was calculated considering each populations couple. Dashed blue line indicates the Fst value observed on the original data (B).


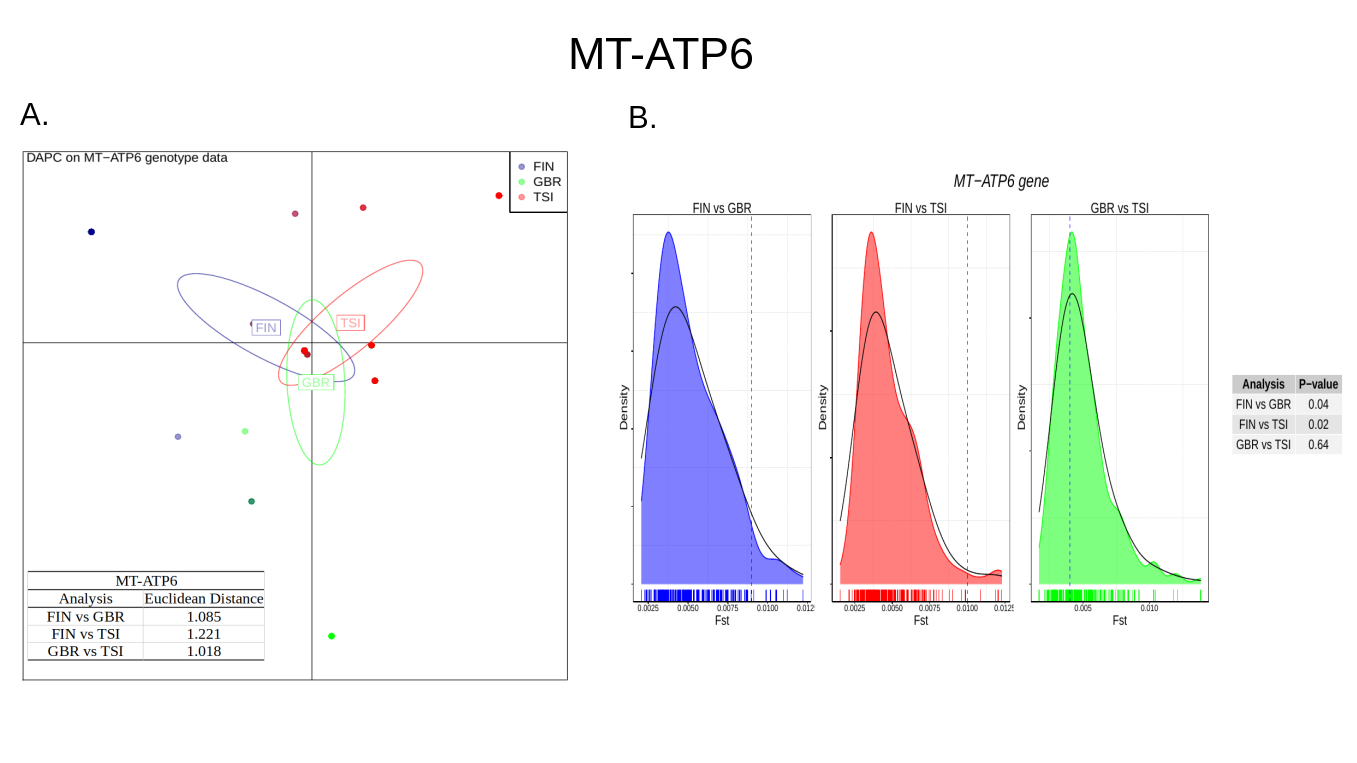


Figure S10: Genetic distance for MT-ATP6 gene considering Finnish (FIN), British (GBR) and Toscani (TSI). DAPC was calculated considering FIN, GBR and TSI (A). FST was calculated considering each populations couple. Dashed blue line indicates the Fst value observed on the original data (B).


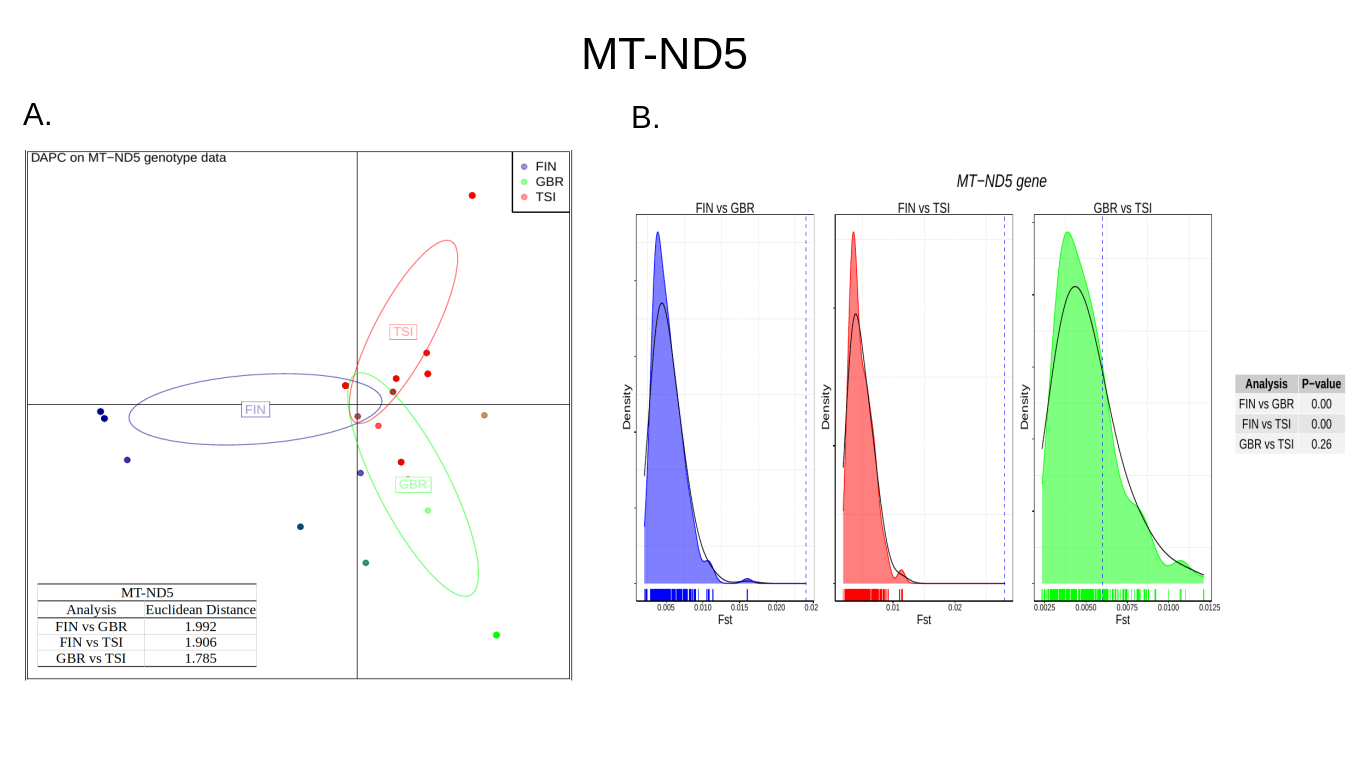


Figure S11: Genetic distance for MT-ND5 gene considering Finnish (FIN), British (GBR) and Toscani (TSI). DAPC was calculated considering FIN, GBR and TSI (A). FST was calculated considering each populations couple. Dashed blue line indicates the Fst value observed on the original data (B).


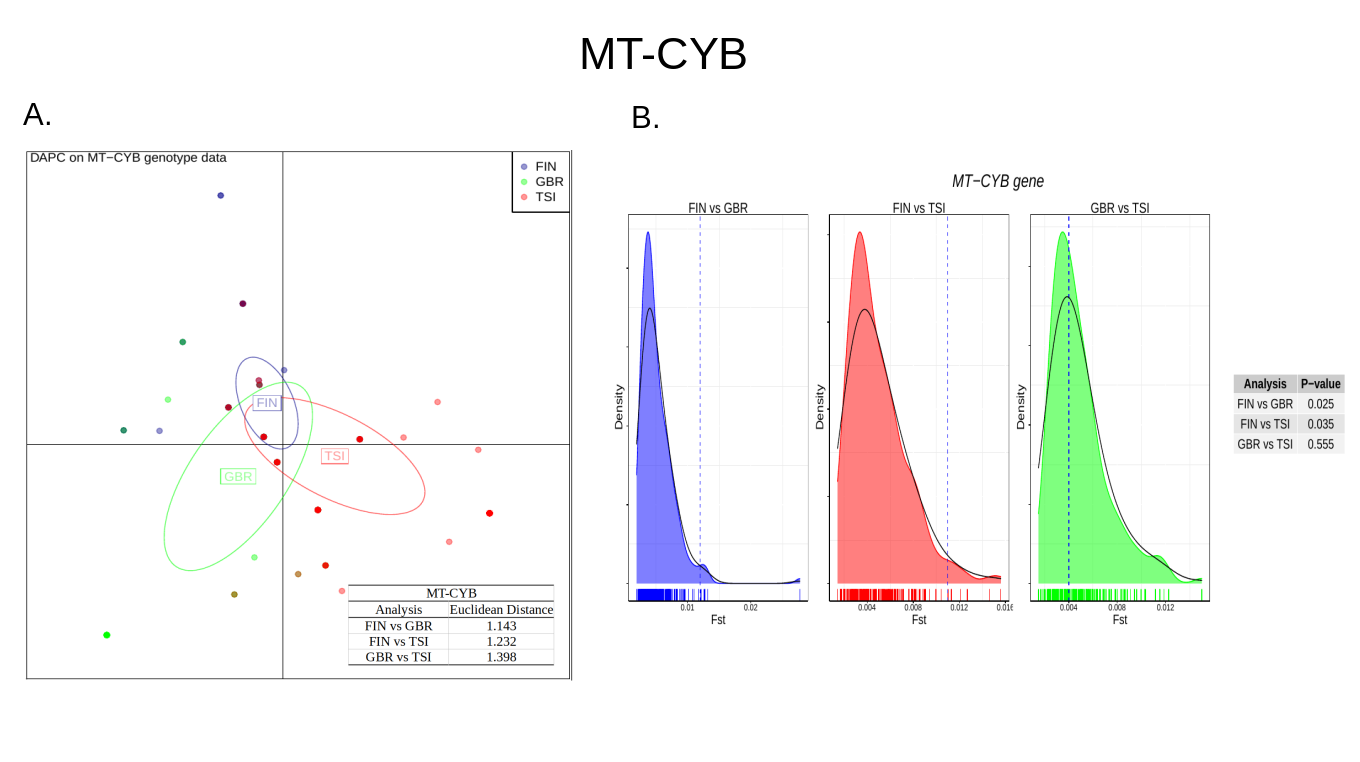


Figure S12: Genetic distance for MT-CYB gene considering Finnish (FIN), British (GBR) and Toscani (TSI). DAPC was calculated considering FIN, GBR and TSI (A). FST was calculated considering each populations couple. Dashed blue line indicates the Fst value observed on the original data (B).


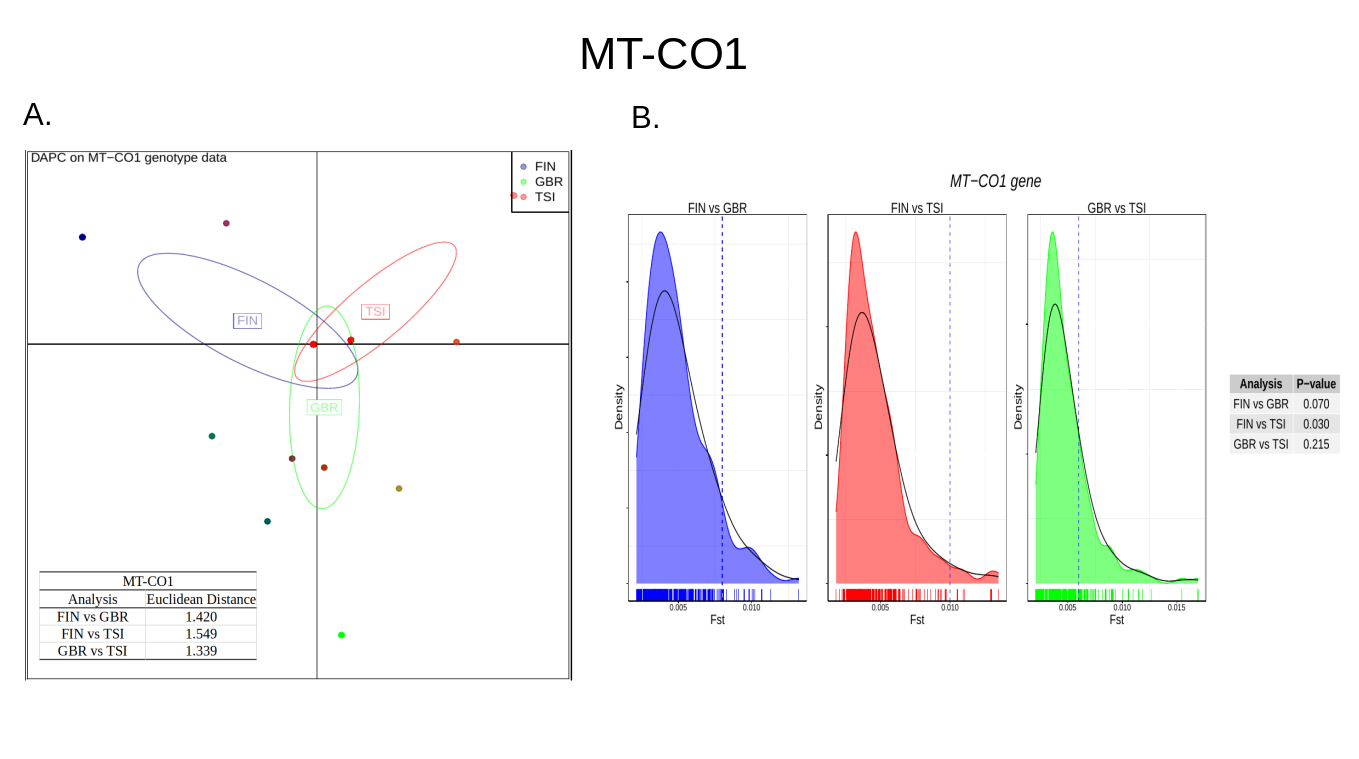


Figure S13: Genetic distance for MT-CO1 gene considering Finnish (FIN), British (GBR) and Toscani (TSI). DAPC was calculated considering FIN, GBR and TSI (A). FST was calculated considering each populations couple. Dashed blue line indicates the Fst value observed on the original data (B).


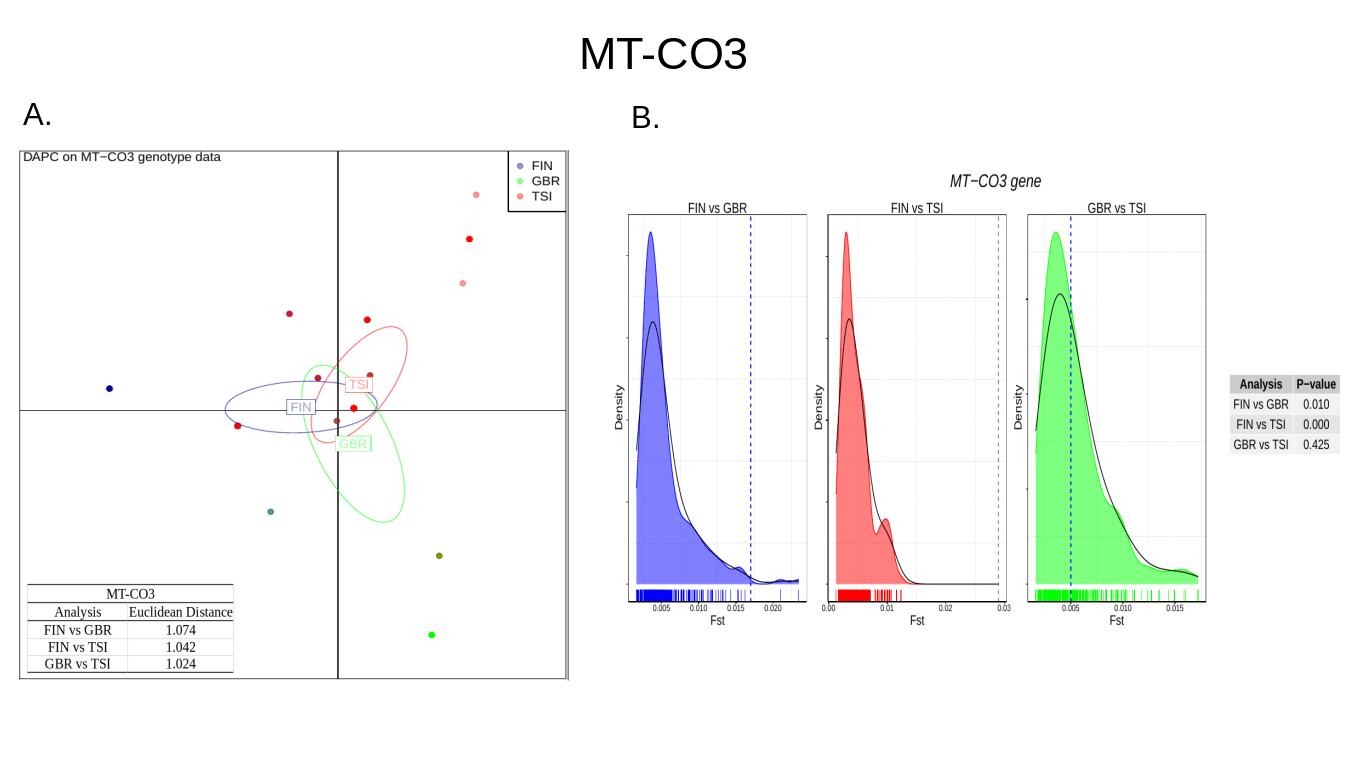


Figure S14: Genetic distance for MT-CO3 gene considering Finnish (FIN), British (GBR) and Toscani (TSI). DAPC was calculated considering FIN, GBR and TSI (A). FST was calculated considering each populations couple. Dashed blue line indicates the Fst value observed on the original data (B).


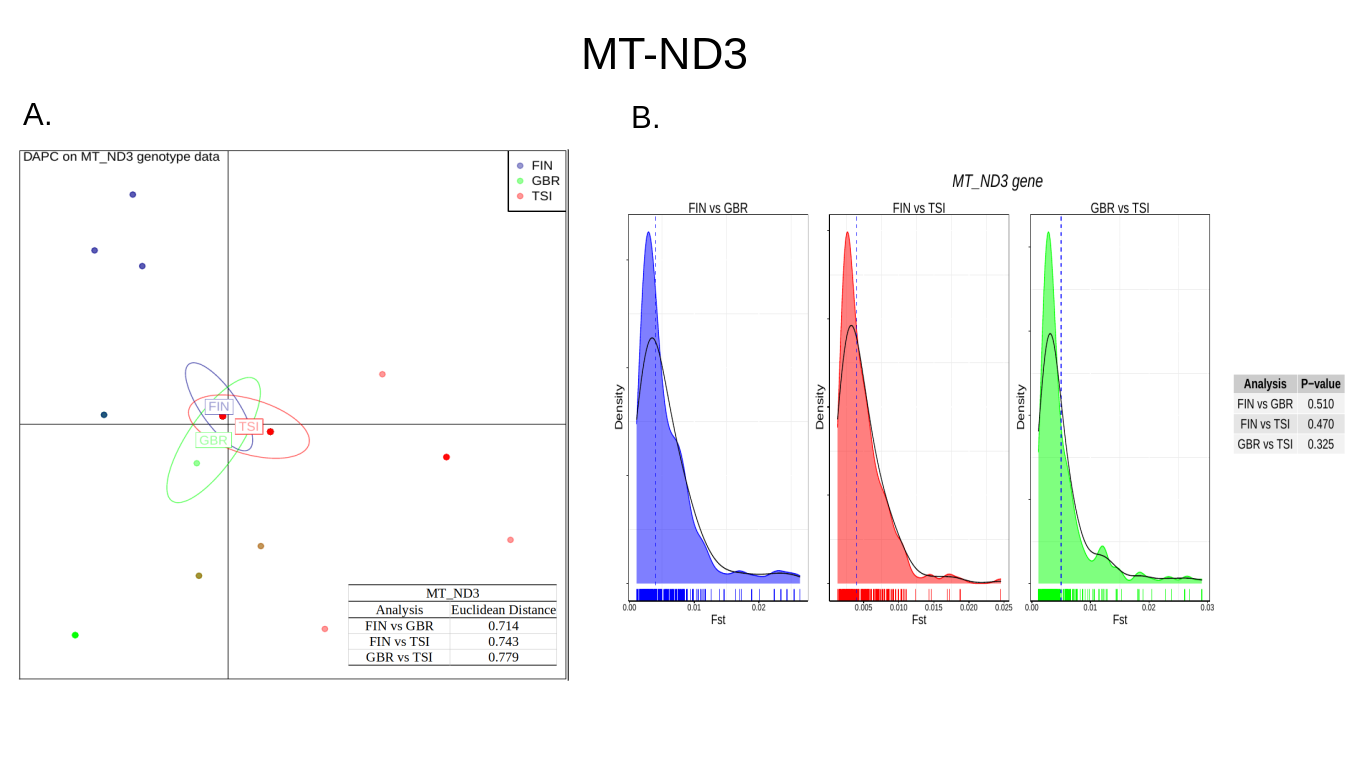


Figure S15: Genetic distance for MT-ND3 gene considering Finnish (FIN), British (GBR) and Toscani (TSI). DAPC was calculated considering FIN, GBR and TSI (A). FST was calculated considering each populations couple. Dashed blue line indicates the Fst value observed on the original data (B).


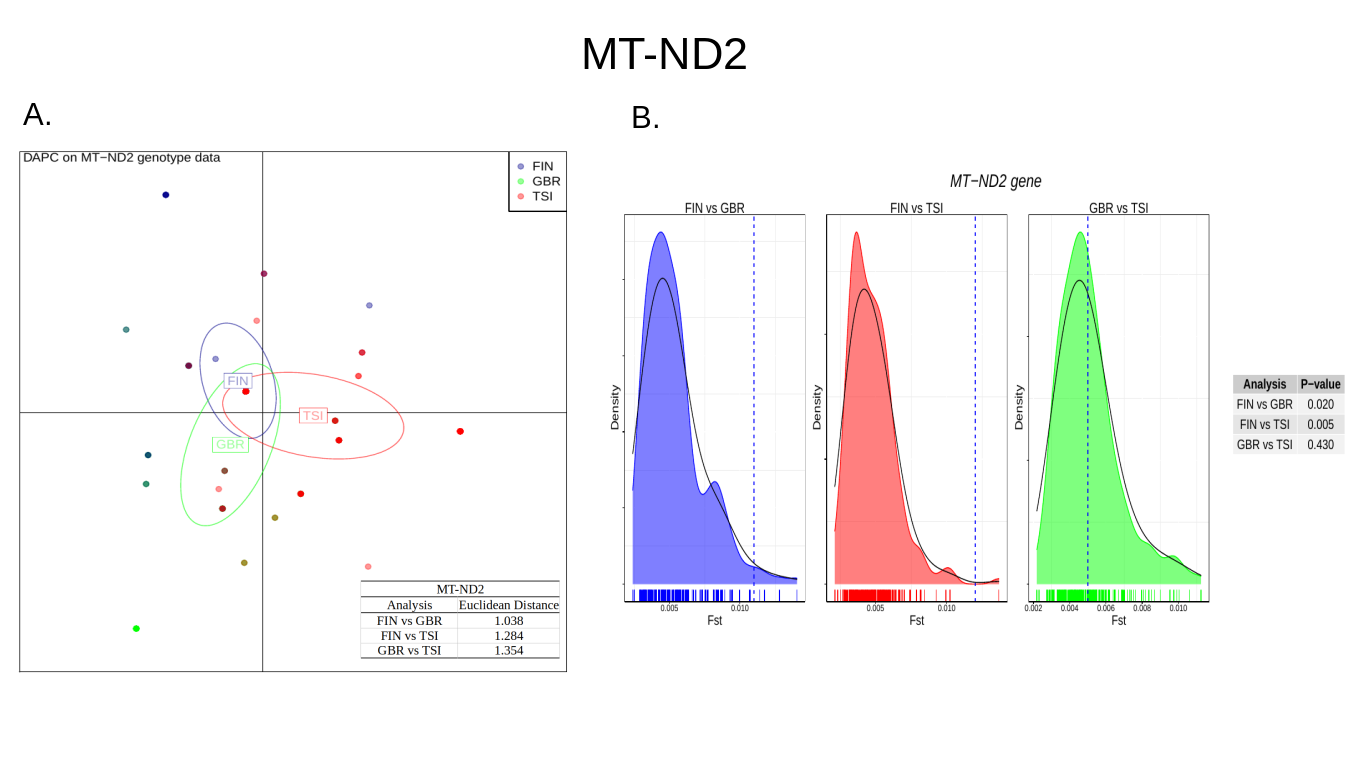


Figure S16: Genetic distance for MT-ND2 gene considering Finnish (FIN), British (GBR) and Toscani (TSI). DAPC was calculated considering FIN, GBR and TSI (A). FST was calculated considering each populations couple. Dashed blue line indicates the Fst value observed on the original data (B).


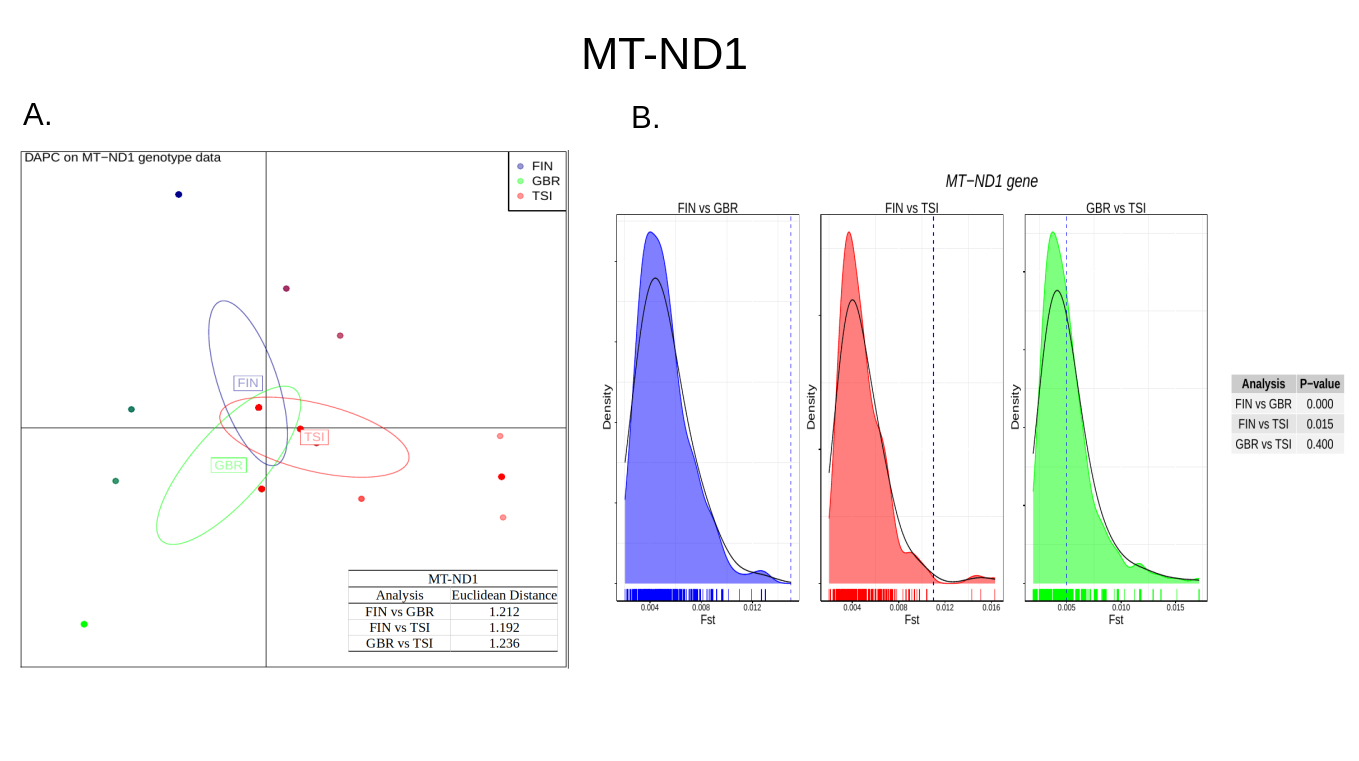


Figure S17: Genetic distance for MT-ND1 gene considering Finnish (FIN), British (GBR) and Toscani (TSI). DAPC was calculated considering FIN, GBR and TSI (A). FST was calculated considering each populations couple. Dashed blue line indicates the Fst value observed on the original data (B).


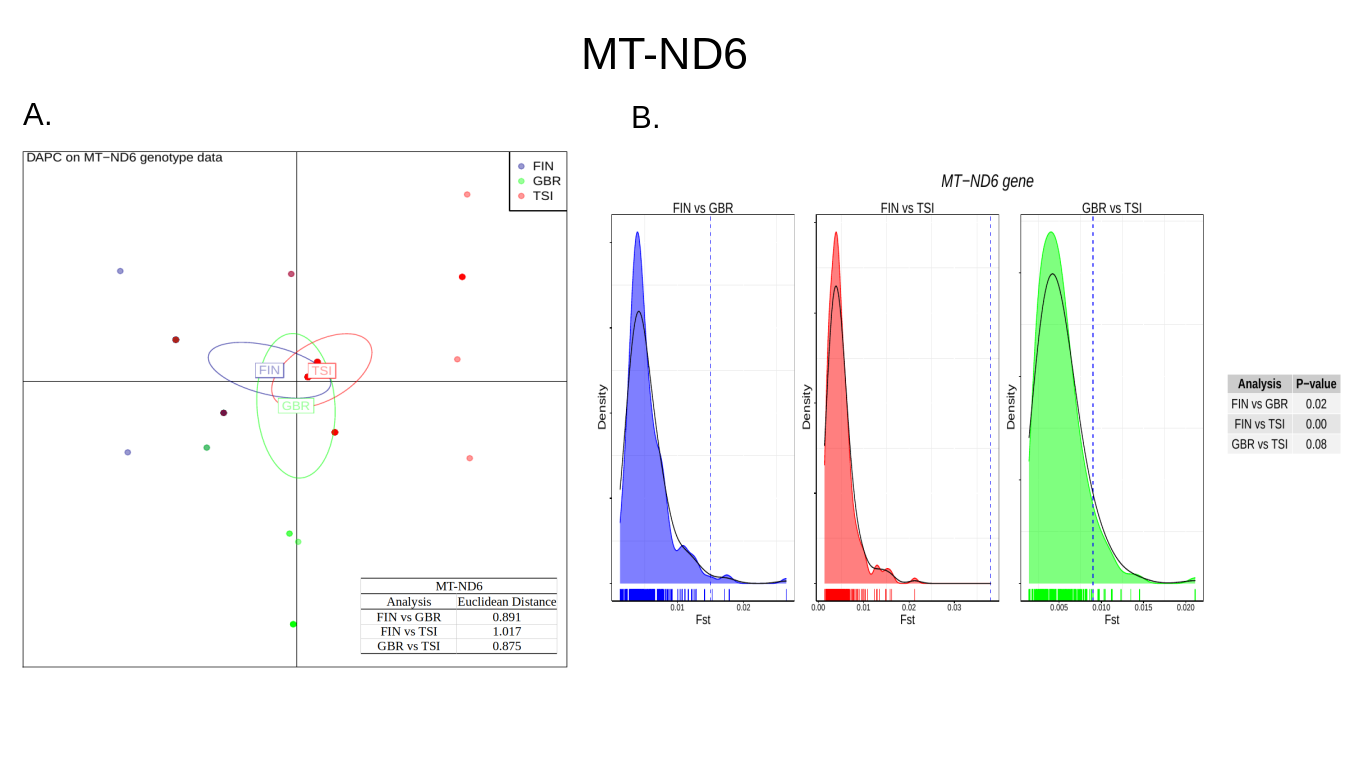


Figure S18: Genetic distance for MT-ND6 gene considering Finnish (FIN), British (GBR) and Toscani (TSI). DAPC was calculated considering FIN, GBR and TSI (A). FST was calculated considering each populations couple. Dashed blue line indicates the Fst value observed on the original data (B).


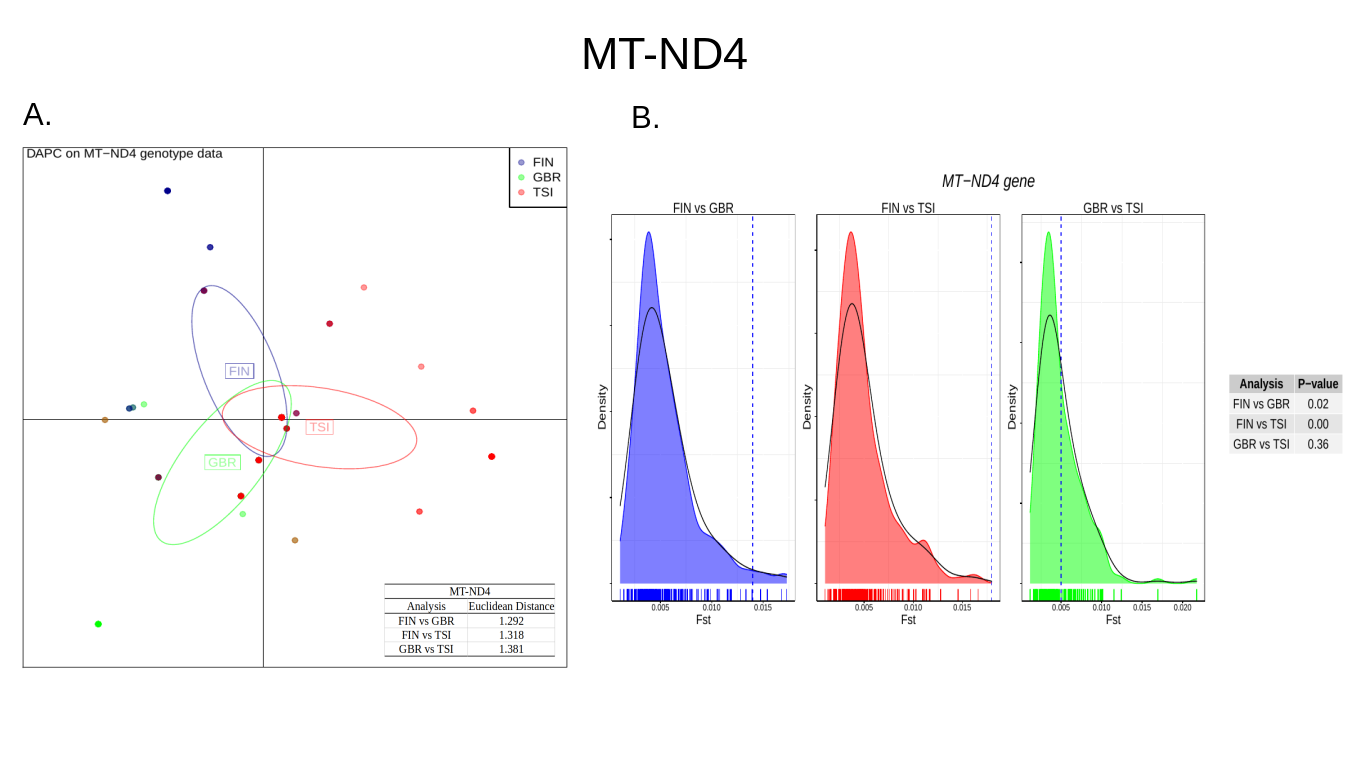


Figure S19: Genetic distance for MT-ND4 gene considering Finnish (FIN), British (GBR) and Toscani (TSI). DAPC was calculated considering FIN, GBR and TSI (A). FST was calculated considering each populations couple. Dashed blue line indicates the Fst value observed on the original data (B).

**SUPPLEMENTARY TABLES**

| NUCLEAR DNA | | | |
| --- | --- | --- | --- |
| Gene | Random Forest | DAPC | Fst |
| PRDM16 | All | All | All |
| LEPR | All | All | All |
| PPARG | FIN | FIN | FIN vs TSI |
| NRF1 | FIN | FIN | FIN |
| UCP3 | TSI | TSI | TSI |
| DIO2 | All | All | FIN |
| PLIN1 | TSI | - | - |
| CIDEA | TSI | All | FIN vs TSI |

Table S1: Genes discriminant role in the 3 analyses. "All": the gene discriminates between all the 3 populations; "TSI": the gene discriminates Toscani from the other two populations; "FIN": the gene discriminates Finnish from the other two populations; "GBR": the gene discriminates British from the other two populations; “FIN vs TSI”: the gene discriminates Finnish from Toscani (and vice versa); -”: the gene has no role in the discrimination.

| MITOCHONDRIAL DNA | | | |
| --- | --- | --- | --- |
| Gene | Random Forest | DAPC | Fst |
| MT-ATP6 | All | FIN | FIN |
| MT-ND5 | All | FIN | FIN |
| MT-CYB | All | - | FIN |
| MT-CO1 | All | FIN vs TSI | FIN vs TSI |
| MT-CO3 | All | - | FIN |
| MT-ND3 | All | - | - |
| MT-ND2 | All | TSI | FIN |
| MT-ND1 | All | - | FIN |
| MT-ND6 | All | FIN vs TSI | FIN |
| MT-ND4 | All | - | FIN |

Table S2: Genes discriminant role in the 3 analyses. "All": the gene discriminates between all the 3 populations; "TSI": the gene discriminates Toscani from the other two populations; "FIN": the gene discriminates Finnish from the other two populations; "GBR": the gene discriminates British from the other two populations; “FIN vs TSI”: the gene discriminates Finnish from Toscani (and vice versa); -”: the gene has no role in the discrimination.

| **Gene** | **References** |
| --- | --- |
| *ADRA1A* | (Strosberg, 1993; Ishida et al., 2018) |
| *ADRB3* | (Jiang et al., 2017) |
| *CIDEA* | (Shimizu and Yokotani, 2009; Jash et al., 2019; Efremova et al., 2020) |
| CREB1 | (Iourgenko et al., 2003) |
| *DIO2* | (Christoffolete et al., 2004; Yau and Yen, 2020) |
| *FTO* | (Claussnitzer et al., 2015) |
| *HOXC4* | (Gesta et al., 2007) |
| *HOXA1* | (Gesta et al., 2007) |
| *LIPE* | (Rosell et al., 2014) |
| *LEP* | (Nikanorova et al., 2020) |
| *LEPR* | (Israel and Chua, 2010) |
| *NRF1* | (Bartelt et al., 2018) |
| *NRIP1* | (Kiskinis et al., 2014; Basse et al., 2015; Deng et al., 2020) |
| *PLIN1* | (Itabe et al., 2017; Hallmark et al., 2019) |
| *PLIN2* | (Yu et al., 2015; Libby et al., 2018) |
| *PLIN3* | (Lee et al., 2018) |
| *PLIN5* | (Barneda et al., 2013; Liang et al., 2019) |
| *PPARG* | (Takenaka et al., 2012) |
| *PPARGC1A* | (Kovaničová et al., 2020) |
| *PPARGC1B* | (Sonoda et al., 2007; Lelliott and Vidal-Puig, 2009) |
| *PRDM16* | (Jankovic et al., 2015; Quagliarello et al., 2017, 16; Efremova et al., 2020) |
| *PRKAR1A* | (McKnight et al., 1998; London et al., 2020) |
| *PRKAR2A* | (McKnight et al., 1998; London et al., 2020) |
| *PRKAR1B* | (McKnight et al., 1998; London et al., 2020) |
| *PRKAR2B* | (McKnight et al., 1998; London et al., 2020) |
| *UCP1* | (Cannon and Nedergaard, 2004; Hancock et al., 2011; Nishimura et al., 2017; Reinisch et al., 2020) |
| *UCP2* | (Caron et al., 2017) |
| *UCP3* | (Hancock et al., 2011; Hilse et al., 2016; Pohl et al., 2019) |

*Table S3: The references which describe the role of the selected genes in cold adaptation, thermoregulation and in brow adipose tissue.*

**References**

Barneda, D., Frontini, A., Cinti, S., and Christian, M. (2013). Dynamic changes in lipid droplet-associated proteins in the “browning” of white adipose tissues. *Biochim. Biophys. Acta BBA - Mol. Cell Biol. Lipids* 1831, 924–933. doi:10.1016/j.bbalip.2013.01.015.

Bartelt, A., Widenmaier, S. B., Schlein, C., Johann, K., Goncalves, R. L. S., Eguchi, K., et al. (2018). Brown adipose tissue thermogenic adaptation requires Nrf1-mediated proteasomal activity. *Nat. Med.* 24, 292–303. doi:10.1038/nm.4481.

Basse, A. L., Dixen, K., Yadav, R., Tygesen, M. P., Qvortrup, K., Kristiansen, K., et al. (2015). Global gene expression profiling of brown to white adipose tissue transformation in sheep reveals novel transcriptional components linked to adipose remodeling. *BMC Genomics* 16, 215. doi:10.1186/s12864-015-1405-8.

Cannon, B., and Nedergaard, J. (2004). Brown Adipose Tissue: Function and Physiological Significance. *Physiol. Rev.* 84, 277–359. doi:10.1152/physrev.00015.2003.

Caron, A., Labbé, S. M., Carter, S., Roy, M.-C., Lecomte, R., Ricquier, D., et al. (2017). Loss of UCP2 impairs cold-induced non-shivering thermogenesis by promoting a shift toward glucose utilization in brown adipose tissue. *Biochimie* 134, 118–126. doi:10.1016/j.biochi.2017.01.006.

Christoffolete, M. A., Linardi, C. C. G., de Jesus, L., Ebina, K. N., Carvalho, S. D., Ribeiro, M. O., et al. (2004). Mice with Targeted Disruption of the Dio2 Gene Have Cold-Induced Overexpression of the Uncoupling Protein 1 Gene but Fail to Increase Brown Adipose Tissue Lipogenesis and Adaptive Thermogenesis. *Diabetes* 53, 577–584. doi:10.2337/diabetes.53.3.577.

Claussnitzer, M., Dankel, S. N., Kim, K.-H., Quon, G., Meuleman, W., Haugen, C., et al. (2015). *FTO* Obesity Variant Circuitry and Adipocyte Browning in Humans. *N. Engl. J. Med.* 373, 895–907. doi:10.1056/NEJMoa1502214.

Deng, J., Guo, Y., Yuan, F., Chen, S., Yin, H., Jiang, X., et al. (2020). Autophagy inhibition prevents glucocorticoid-increased adiposity via suppressing BAT whitening. *Autophagy* 16, 451–465. doi:10.1080/15548627.2019.1628537.

Efremova, A., Colleluori, G., Thomsky, M., Perugini, J., Protasoni, M., Reguzzoni, M., et al. (2020). Biomarkers of Browning in Cold Exposed Siberian Adults. *Nutrients* 12, 2162. doi:10.3390/nu12082162.

Gesta, S., Tseng, Y.-H., and Kahn, C. R. (2007). Developmental Origin of Fat: Tracking Obesity to Its Source. *Cell* 131, 242–256. doi:10.1016/j.cell.2007.10.004.

Hallmark, B., Karafet, T. M., Hsieh, P., Osipova, L. P., Watkins, J. C., and Hammer, M. F. (2019). Genomic Evidence of Local Adaptation to Climate and Diet in Indigenous Siberians. *Mol. Biol. Evol.* 36, 315–327. doi:10.1093/molbev/msy211.

Hancock, A. M., Witonsky, D. B., Alkorta-Aranburu, G., Beall, C. M., Gebremedhin, A., Sukernik, R., et al. (2011). Adaptations to climate-mediated selective pressures in humans. *PLoS Genet.* 7, e1001375. doi:10.1371/journal.pgen.1001375.

Hilse, K. E., Kalinovich, A. V., Rupprecht, A., Smorodchenko, A., Zeitz, U., Staniek, K., et al. (2016). The expression of UCP3 directly correlates to UCP1 abundance in brown adipose tissue. *Biochim. Biophys. Acta BBA - Bioenerg.* 1857, 72–78. doi:10.1016/j.bbabio.2015.10.011.

Iourgenko, V., Zhang, W., Mickanin, C., Daly, I., Jiang, C., Hexham, J. M., et al. (2003). Identification of a family of cAMP response element-binding protein coactivators by genome-scale functional analysis in mammalian cells. *Proc. Natl. Acad. Sci.* 100, 12147–12152. doi:10.1073/pnas.1932773100.

Ishida, H., Saito, S., and Ishikawa, T. (2018). α1A-Adrenoceptors, but not α1B- or α1D-adrenoceptors, contribute to enhanced contractile response to phenylephrine in cooling conditions in the rat tail artery. *Eur. J. Pharmacol.* 838, 120–128. doi:10.1016/j.ejphar.2018.09.004.

Israel, D., and Chua, S. (2010). Leptin receptor modulation of adiposity and fertility. *Trends Endocrinol. Metab.* 21, 10–16. doi:10.1016/j.tem.2009.07.004.

Itabe, H., Yamaguchi, T., Nimura, S., and Sasabe, N. (2017). Perilipins: a diversity of intracellular lipid droplet proteins. *Lipids Health Dis.* 16, 83. doi:10.1186/s12944-017-0473-y.

Jankovic, A., Golic, I., Markelic, M., Stancic, A., Otasevic, V., Buzadzic, B., et al. (2015). Two key temporally distinguishable molecular and cellular components of white adipose tissue browning during cold acclimation: Browning of white adipose tissue during cold acclimation. *J. Physiol.* 593, 3267–3280. doi:10.1113/JP270805.

Jash, S., Banerjee, S., Lee, M.-J., Farmer, S. R., and Puri, V. (2019). CIDEA Transcriptionally Regulates UCP1 for Britening and Thermogenesis in Human Fat Cells. *iScience* 20, 73–89. doi:10.1016/j.isci.2019.09.011.

Jiang, Y., Berry, D. C., and Graff, J. M. (2017). Distinct cellular and molecular mechanisms for β3 adrenergic receptor-induced beige adipocyte formation. *eLife* 6, e30329. doi:10.7554/eLife.30329.

Kiskinis, E., Chatzeli, L., Curry, E., Kaforou, M., Frontini, A., Cinti, S., et al. (2014). RIP140 Represses the “Brown-in-White” Adipocyte Program Including a Futile Cycle of Triacyclglycerol Breakdown and Synthesis. *Mol. Endocrinol.* 28, 344–356. doi:10.1210/me.2013-1254.

Kovaničová, Z., Kurdiová, T., Baláž, M., Štefanička, P., Varga, L., Kulterer, O. C., et al. (2020). Cold Exposure Distinctively Modulates Parathyroid and Thyroid Hormones in Cold-Acclimatized and Non-Acclimatized Humans. *Endocrinology* 161, bqaa051. doi:10.1210/endocr/bqaa051.

Lee, Y. K., Sohn, J. H., Han, J. S., Park, Y. J., Jeon, Y. G., Ji, Y., et al. (2018). *Perilipin 3* Deficiency Stimulates Thermogenic Beige Adipocytes Through *PPARα* Activation. *Diabetes* 67, 791–804. doi:10.2337/db17-0983.

Lelliott, C. J., and Vidal-Puig, A. (2009). “PGC-1β: A Co-activator That Sets the Tone for Both Basal and Stress-Stimulated Mitochondrial Activity,” in *Early Nutrition Programming and Health Outcomes in Later Life* Advances in Experimental Medicine and Biology., eds. B. Koletzko, T. Decsi, D. Molnár, and A. de la Hunty (Dordrecht: Springer Netherlands), 133–139. doi:10.1007/978-1-4020-9173-5_15.

Liang, X., Pan, J., Cao, C., Zhang, L., Zhao, Y., Fan, Y., et al. (2019). Transcriptional Response of Subcutaneous White Adipose Tissue to Acute Cold Exposure in Mice. *Int. J. Mol. Sci.* 20, 3968. doi:10.3390/ijms20163968.

Libby, A. E., Bales, E. S., Monks, J., Orlicky, D. J., and McManaman, J. L. (2018). Perilipin-2 deletion promotes carbohydrate-mediated browning of white adipose tissue at ambient temperature. *J. Lipid Res.* 59, 1482–1500. doi:10.1194/jlr.M086249.

London, E., Bloyd, M., and Stratakis, C. A. (2020). PKA functions in metabolism and resistance to obesity: lessons from mouse and human studies. *J. Endocrinol.* 246, R51–R64. doi:10.1530/JOE-20-0035.

McKnight, G. S., Cummings, D. E., Amieux, P. S., Sikorski, M. A., Brandon, E. P., Planas, J. V., et al. (1998). Cyclic AMP, PKA, and the physiological regulation of adiposity. *Recent Prog. Horm. Res.* 53, 139–159; discussion 160-161.

Nikanorova, A. A., Barashkov, N. A., Nakhodkin, S. S., Pshennikova, V. G., Solovyev, A. V., Romanov, G. P., et al. (2020). The Role of Leptin Levels in Adaptation to Cold Climates. *Int. J. Environ. Res. Public. Health* 17, 1854. doi:10.3390/ijerph17061854.

Nishimura, T., Katsumura, T., Motoi, M., Oota, H., and Watanuki, S. (2017). Experimental evidence reveals the UCP1 genotype changes the oxygen consumption attributed to non-shivering thermogenesis in humans. *Sci. Rep.* 7, 5570. doi:10.1038/s41598-017-05766-3.

Pohl, E. E., Rupprecht, A., Macher, G., and Hilse, K. E. (2019). Important Trends in UCP3 Investigation. *Front. Physiol.* 10, 470. doi:10.3389/fphys.2019.00470.

Quagliarello, A., De Fanti, S., Giuliani, C., Abondio, P., Sarno, S., Sazzini, M., et al. (2017). Multiple selective events at the PRDM16 functional pathway shaped adaptation of western European populations to different climate conditions. *J. Anthropol. Sci.*, 235–247. doi:10.4436/JASS.95011.

Reinisch, I., Schreiber, R., and Prokesch, A. (2020). Regulation of thermogenic adipocytes during fasting and cold. *Mol. Cell. Endocrinol.* 512, 110869. doi:10.1016/j.mce.2020.110869.

Rosell, M., Kaforou, M., Frontini, A., Okolo, A., Chan, Y.-W., Nikolopoulou, E., et al. (2014). Brown and white adipose tissues: intrinsic differences in gene expression and response to cold exposure in mice. *Am. J. Physiol.-Endocrinol. Metab.* 306, E945–E964. doi:10.1152/ajpendo.00473.2013.

Shimizu, T., and Yokotani, K. (2009). Acute cold exposure-induced down-regulation of CIDEA, cell death-inducing DNA fragmentation factor-α-like effector A, in rat interscapular brown adipose tissue by sympathetically activated β3-adrenoreceptors. *Biochem. Biophys. Res. Commun.* 387, 294–299. doi:10.1016/j.bbrc.2009.06.147.

Sonoda, J., Mehl, I. R., Chong, L.-W., Nofsinger, R. R., and Evans, R. M. (2007). PGC-1beta controls mitochondrial metabolism to modulate circadian activity, adaptive thermogenesis, and hepatic steatosis. *Proc. Natl. Acad. Sci.* 104, 5223–5228. doi:10.1073/pnas.0611623104.

Strosberg, A. D. (1993). Structure, function, and regulation of adrenergic receptors. *Protein Sci.* 2, 1198–1209. doi:10.1002/pro.5560020802.

Takenaka, A., Nakamura, S., Mitsunaga, F., Inoue-Murayama, M., Udono, T., and Suryobroto, B. (2012). Human-Specific SNP in Obesity Genes, Adrenergic Receptor Beta2 (ADRB2), Beta3 (ADRB3), and PPAR γ2 (PPARG), during Primate Evolution. *PLoS ONE* 7, e43461. doi:10.1371/journal.pone.0043461.

Yau, W. W., and Yen, P. M. (2020). Thermogenesis in Adipose Tissue Activated by Thyroid Hormone. *Int. J. Mol. Sci.* 21, 3020. doi:10.3390/ijms21083020.

Yu, J., Zhang, S., Cui, L., Wang, W., Na, H., Zhu, X., et al. (2015). Lipid droplet remodeling and interaction with mitochondria in mouse brown adipose tissue during cold treatment. *Biochim. Biophys. Acta BBA - Mol. Cell Res.* 1853, 918–928. doi:10.1016/j.bbamcr.2015.01.020.
